# Experimental Studies and Dynamics Modeling Analysis of the Swimming and Diving of Whirligig Beetles (*Coleoptera:* *Gyrinidae*)

**DOI:** 10.1371/journal.pcbi.1002792

**Published:** 2012-11-29

**Authors:** Zhonghua Xu, Scott C. Lenaghan, Benjamin E. Reese, Xinghua Jia, Mingjun Zhang

**Affiliations:** Department of Mechanical, Aerospace, and Biomedical Engineering, University of Tennessee, Knoxville, Tennessee, United States of America; Carnegie Mellon University, United States of America

## Abstract

Whirligig beetles (*Coleoptera*, *Gyrinidae*) can fly through the air, swiftly swim on the surface of water, and quickly dive across the air-water interface. The propulsive efficiency of the species is believed to be one of the highest measured for a thrust generating apparatus within the animal kingdom. The goals of this research were to understand the distinctive biological mechanisms that allow the beetles to swim and dive, while searching for potential bio-inspired robotics applications. Through static and dynamic measurements obtained using a combination of microscopy and high-speed imaging, parameters associated with the morphology and beating kinematics of the whirligig beetle's legs in swimming and diving were obtained. Using data obtained from these experiments, dynamics models of both swimming and diving were developed. Through analysis of simulations conducted using these models it was possible to determine several key principles associated with the swimming and diving processes. First, we determined that curved swimming trajectories were more energy efficient than linear trajectories, which explains why they are more often observed in nature. Second, we concluded that the hind legs were able to propel the beetle farther than the middle legs, and also that the hind legs were able to generate a larger angular velocity than the middle legs. However, analysis of circular swimming trajectories showed that the middle legs were important in maintaining stable trajectories, and thus were necessary for steering. Finally, we discovered that in order for the beetle to transition from swimming to diving, the legs must change the plane in which they beat, which provides the force required to alter the tilt angle of the body necessary to break the surface tension of water. We have further examined how the principles learned from this study may be applied to the design of bio-inspired swimming/diving robots.

## Author Summary

The whirligig beetles belong to the family Gyrinidae, consisting of over 700 species of water beetles. They are characterized by a divided eye, ellipsoidal body, and rapidly swim in circles when alarmed. Perhaps the most interesting characteristic of whirligig beetles is their ability to rapidly swim on the surface of water, and also to quickly transition to diving beneath the surface. In this study, we have measured the key physical parameters that allow whirligig beetles to swim and dive, and have used these values to develop dynamics models of the swimming and diving processes. Based on these models, we were able to analyze how the beetle is capable of making sharp turns, the efficiency of varying leg beating patterns, and the key parameters involved in swimming, as well as diving. We were then able to identify fundamental principles used by the beetle to transition from swimming to diving, and examine how the morphology and “design” of the beetle leads to its ability to rapidly swim and maneuver. Based on the results obtained, we further identified principles and components of the beetle design that could be translated into the development of bio-inspired robotics.

## Introduction

Few organisms maintain the ability to freely crawl on land, swim in water, and fly through the air; however, the whirligig beetle (*Coleoptera Gyrinidae*) is able to efficiently maneuver in all three environments [1]. The whirligig beetle also has the fastest measured speed for a swimming insect, while still maintaining the ability to produce very sharp turns. In this study, we will focus on investigating how the whirligig beetle uses its legs to swim on the surface of water, and how it transitions from surface swimming to diving. Ultimately, we will use mathematical models combined with experimental data to quantitatively characterize the detailed kinematics and dynamics for the swimming and diving processes.

**Figure 1:**
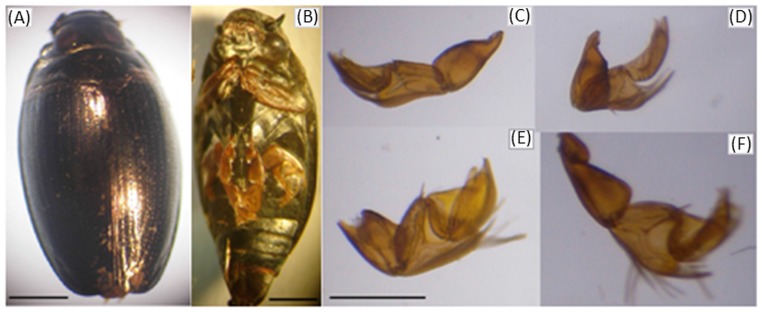
Light micrographs of the whirligig beetle. (A) Dorsal view of the beetle, demonstrating the overall shape. (B) Ventral view of the beetle showing the fore, middle and hind legs. (C&D) Micrographs of dissected middle right (C) and left (D) legs. (E&F) Micrographs of dissected hind right (E) and left (F) legs. Measurements of leg length (*L_h_* and *L_m_*) and area (*S_h−_* and *S_m−_*) were made from micrographs of dissected legs. The scale bars are 1 mm.

The morphology of whirligig beetles is highly adapted for the environment in which they live. As shown in **Figure 1A–B**, they have divided compound eyes for simultaneously looking above and below the water's surface, a pronounced pair of anterior appendages for grasping prey and climbing, and two pairs of paddle-like legs for swimming [2]. While many studies have attempted to understand the highly efficient swimming motion of whirligig beetles [2], [3], [4], few studies have investigated the swimming mechanism and the transition to diving. The insect's ability to swiftly transition between swimming and diving is particularly interesting for bio-inspiration of swimming/diving robots. In this paper, parameters related to swimming and diving of the whirligig beetle were characterized through experimental analysis, and further used to conduct simulations to answer questions that could not be experimentally verified.

Water-walking arthropods have received much attention in recent years [5], [6], [7], [8], [9], [10], [11], [12], [13], however, less research has been conducted on swimming/diving insects. Despite this fact, whirligig beetles have long fascinated researchers, owing to some of the astounding features of their movement. Previous studies have concluded that whirligig beetles can swim at speeds up to 44.5 body lengths/s with a maximum turning rate of 4428°/s and a maximum centripetal acceleration of 2.86 g [14]. In addition to the incredible speed these insects are able to achieve, the turning radius can be as small as 24% of the body length, and typically 84% of the energy devoted to swimming can be transformed into forward propulsion [1], [15]. This propulsive efficiency is believed to be the highest measured for a thrust generating apparatus within the animal kingdom [3]. Additionally, the swimming mechanics of the whirligig beetle has been studied in terms of wave management and turning performance [1], [4], [16], [17]. Findings from these studies have also shown that whirligig beetles are able to attain high swimming speeds while reducing or avoiding hydroplaning and maximum drag due to their unique leg kinematics and structures. Results from studying the management of fluid and wave resistances produced by the beetles, has also led to a better understanding of the efficiency of their propulsive mechanisms, and what enables the insect to maintain such high speeds. In order to understand the ability of the insect to rapidly and efficiently maneuver, it is necessary to investigate how it uses the unique morphology of its propulsive structures to swim and dive.

One of the morphological adaptations that allow whirligig beetles to rapidly swim on the surface of water is the streamlined ellipsoidal body shape, which minimizes the fluid resistance [14]. The forward propulsive efficiency is further increased by the prevention of lateral movement due to the rigid body of the beetle. Although lateral forces do not make direct contributions to forward propulsion, they assist in this process by increasing stability and maneuverability [18]. To maintain this low drag body shape, the forelegs remain folded underneath the body, preventing drag that would be generated, if they were extended [1]. To further reduce the drag when moving on the surface, the beetle has been reported to have a waxy covering that prevents wetting of the body [19], [20]. But perhaps the most important morphological characteristic of the whirligig beetle in relation to its propulsive efficiency is the design of the swimming legs. Unlike the forelegs, the middle and hind legs have evolved into highly efficient swimming paddles with specialized morphology [3]. As shown in **Figure 1C–F**, both pairs of the swimming legs, termed middle and hind legs, have a large number of swimming “hairs” that increase the effective contact area generating a larger propulsive force [21]. During the power phase, the middle and hind legs have a contact area about 40 times greater than during the recovery phase [3], [15]. A previous report indicated that the middle legs can also paddle at a frequency up to 25 Hz, with the hind legs beating twice as fast [22]. When the beetle swims in a straight line, the left and right swimming legs beat together with the hind and middle legs beating in an alternating fashion. However, the left and right legs paddle asymmetrically during turning [1].

Another feature of the whirligig beetles' unique motion is its ability to rapidly transition from swimming on the water surface to diving below the surface. While the diving behavior has been observed as a necessary trait for predator avoidance and egg laying, the dynamics associated with the transition from swimming to diving has not been well understood. In fact, the effects of swimming acceleration and body size on the mechanical energy consumptions of diving have only been investigated in a few organisms, such as ducks [23] and marine mammals [24], [25]. In general, the rigid exoskeleton of insects limits the efficiency of diving. In this paper, we will examine how the whirligig beetle can overcome this limitation.

This research combines both experimental analysis of the swimming and diving of the whirligig beetles, and the development of dynamics models to better understand these behaviors. By using parameters obtained from the systematic analysis of high-speed video and microscopic images, dynamics models for both the swimming and diving patterns were developed. Based on simulations from these models, we were able to understand several phenomena that could not be directly observed through experimental studies and further inspire principles that may be used in the design of swimming or diving robots.

## Results/Discussion

### Swimming parameters obtained from imaging and high-speed video experiments

**Figure 2:**
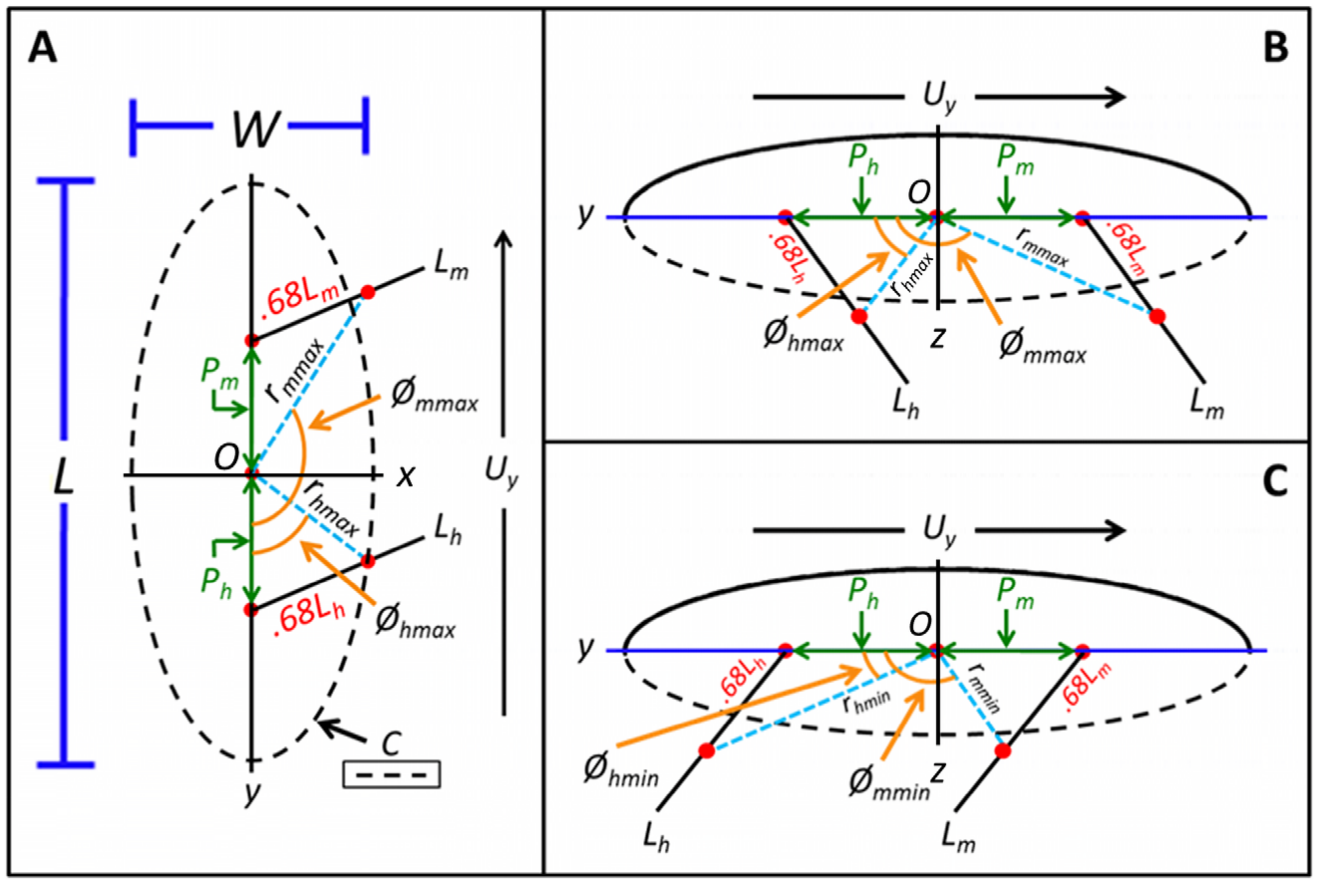
Diagram demonstrating how each parameter was calculated. (A) Top-down view of the body showing key parameters for swimming. (B&C) Side view of the body on the surface of water (indicated by blue line) showing both the maximum and minimum position of the legs during a leg beat when diving. In all of the above diagrams, the hind legs are indicated by the subscript *h*, while the middle legs are indicated by the subscript *m*. Using this notation, the length of the hind legs is *L_h_*, etc. The direction of motion of the beetle is indicated by an arrow showing the forward velocity (*U_y_*). The dashed line in B&C indicates the submerged portion of the beetle. All other parameters designations are listed in **Tables 1** **–** [Table pcbi-1002792-t002] **3**.

In order to build dynamics models for both swimming and diving, it was necessary to determine the parameters related to the dimensions of whirligig beetles. The average beetles' mass (*M*) was determined to be 10±2 mg by blotting the beetles with filter paper and weighing them on a precision balance. To determine the morphology of the beetles, images were captured while they were floating on the water's surface. Measurements obtained from feature traces were conducted in *ImageJ*. From the acquired traces, we identified that the beetles had a characteristic body length (*L_b_*) of 5.23±0.31 mm and a width (*W_b_*) of 2.2±0.23 mm, as shown in **Figure 2A**. Additionally, by tracing the outline of the beetle's body, the contact line length (*C*) was determined to be 10.93±0.54 mm, and the contact area (*S_b_*) was 7.32±0.14 mm^2^. Using a similar tracing approach, the depth of the submerged portion of the body (*h*) was determined to be 0.74±0.14 mm. The average frontal area (*A_y_*) and average side area (*A_x_*) of the beetles in contact with water was calculated by tracing the submerged portion of the body in the *y*–*z* and *x*–*z* planes as shown in **Figure 2B–C**, and was determined to be 1.31±0.4 mm^2^ and 2.65±0.2 mm^2^, respectively. For the convenience of analysis, we define the *x*–*y*–*z* coordinates as the lateral direction (*x*-axis), the longitudinal direction (*y*-axis, the forward direction), and the vertical direction (*z*-axis) (**Figures S1 & S2**). Due to the small size of the legs, it was necessary to use a higher magnification imaging system to determine the accurate measurements of their morphology.

**Figure 3:**
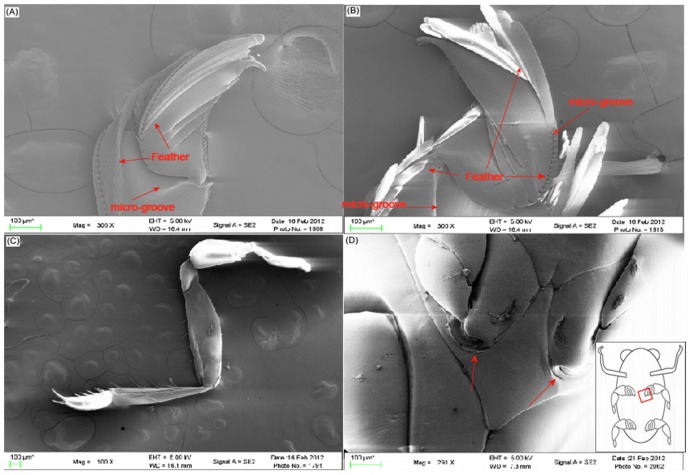
The SEM micrographs of the legs of *Gyrinus*. (A) SEM micrograph of the middle leg showing the folded swimming laminae. On the middle leg, the laminae are predominately on the outer surface. (B) SEM micrograph of the hind leg demonstrating the presence of laminae on both the inner and outer surface of the rowing blade. (C) SEM micrograph showing the significantly altered morphology of the foreleg. (D) Image of the point of attachment of a leg. The inset demonstrates the location of the micrograph relative to the beetle's body, with the area analyzed highlighted by the red box. SEM micrographs were used to measure the length (*L_laminae_*) and width (*W_laminae_*) of the laminae for calculation of the effective are of the hind (*S_h+_*) and middle legs (*S_m+_*) with laminae extended. In all micrographs, the scale bar = 100 µm.

As shown in **Figure 1C–F**, light micrographs of dissected middle and rear legs were analyzed to obtain dimensions for these structures. From the micrographs, the length of the middle legs (*L_m_*) was 2.08±0.08 mm, and the length of the hind legs was (*L_h_*) 2.67±0.03 mm. By conducting polygonal traces of the hind legs when they were outstretched, we have obtained the area of these legs without the swimming laminae extended (S*_h−_*) as 1.08±0.03 mm^2^. Similarly, analysis of the middle legs showed a reduced area (*S_m−_*) of only 0.66±0.01 mm^2^. Considering that the true effective area of the swimming legs during the power stroke is dependent on the extension of “swimming laminae” used to increase the surface area [26], it was necessary to measure the increase in area with these structures extended. Due to the small size of the laminae, scanning electron microscopy (SEM) was used to measure the size of these structures, as shown in **Figure 3**. Based on the SEM data, the average length of the laminae (*L_laminae_*) was 366.98±31.3 µm with an average width (*W_laminae_*) of 30.84±0.03 µm. It appeared that the laminae on the exterior portion of the leg were longer than those on the interior portion of the legs. Previous studies of *Gyrinus* indicated that 74 laminae were present on the hind legs and 47 on the middle legs [26], [27]. Using this value for the number of laminae, the effective area of the hind legs with the laminae extended (*S_h+_*) was estimated to be 1.92 mm^2^. This represents a 77% increase in propulsive area compared to the hind leg without the laminae extended. The area for the middle legs with the laminae extended (S_m+_) was 1.19 mm^2^, representing an 80% increase in propulsive area, when compared to the folded state. Since the laminae are folded in the recovery phase of the beat and only extended in the power phase, this leads to a reduced-drag recovery stroke aiding in the propulsive efficiency. On the other hand, the legs may also be oriented at different angles, so that the maximum area is not perpendicular to the direction in which the beetle is moving. All parameter values obtained from the analysis of the imaging data are summarized in **Table 1**.

**Table 1:** Parameters obtained from micrographs.

**M**	Mass of beetle	10.0±2 mg
***L_b_***	Average body length	5.23±0.31 mm
**W_b_**	Average body width	2.2±0.23 mm
**H_b_**	Average body height	1.40±0.18 mm
***L_h_***	Average length of hind legs	2.67±0.03 mm
***L_m_***	Average length of middle legs	2.08±0.08 mm
**C**	Length of the contact line of the body with water	10.93±0.54 mm
**S_b_**	Area of body in contact with the water	7.32±0.14 mm^2^
***h***	Average depth of submerged portion of the body	0.74±0.08 mm
**S_h+_**	Effective area of the hind legs with laminae extended	1.92 mm^2^
**S_h−_**	Effective area of the hind legs without laminae extended	1.08 mm^2^±0.03 mm^2^
**S_m+_**	Effective area of the middle legs with laminae extended	1.19 mm^2^
**S_m−_**	Effective area of the middle legs without laminae extended	0.66±0.01 mm^2^
**L_laminae_**	Average length of laminae	366.98±31.3 µm
**W_laminae_**	Average width of laminae	30.84±10.5 µm
**A_y_**	Average frontal area of the beetle	1.31±0.4 mm^2^
**A_x_**	Average side area of beetle	2.65±0.2 mm^2^
***P_m_***	Distance from point of attachment of middle leg to origin	0.44 mm
***P_h_***	Distance from point of attachment of hind leg to origin	0.53 mm

**Figure 4:**
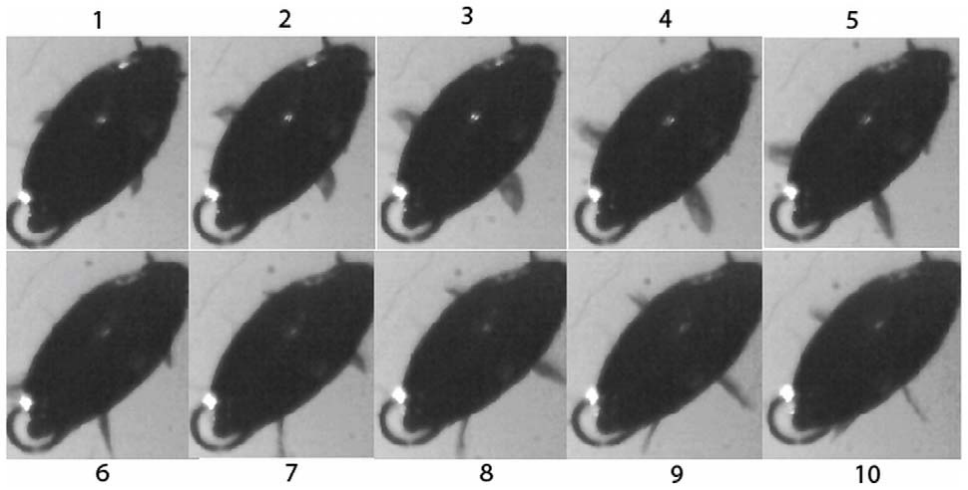
The sequence of one hind leg stroke. In frames 1–5, only the hind leg is visible, with the middle leg emerging in frame 6. In frames 6–10 it is possible to observe the beating of both legs. During the course of one leg stroke, the effective area of the legs decreases in the horizontal plane, indicating that the effective area for forward propelling increases.

After completing analysis of the static images, experimental measurements were obtained from the high-speed camera system in order to obtain parameters related to the dynamic motion of the beetle. A typical hind leg stroke is shown in **Figure 4** and **Video S1**. Based on our experimental studies, we observed a peak leg speed in the forward direction (*U_p_*) of 0.67 m/s for rapid swimming. In addition, the average forward velocity (*U_y_*) of the beetles observed in this study was 0.0936±0.0226 m/s. The maximum forward velocity (*U_max_*) was 0.8 m/s, which is astonishing for such a small organism. The maximum forward speed (*U_max_*) measured is in agreement with that reported by Voise [1]. Important parameters related to the development of the dynamics model for swimming were the values related to the beating motion of the swimming legs, and their positions relative to the center of mass of the beetle. The center of mass of the beetle was assumed to be the center point of the beetle on the *x*–*y* axis. Previous studies have shown that the legs during one beating cycle have a maximum sweep of ∼120° around the point of attachment of the leg [3]. To determine the maximum and minimum angles between the negative *y* axis and the straight line from the center of mass to the acting point of force on the legs, the maximum and minimum angles of both the hind (*Ø_hmax_*,, *Ø_hmin_*) and middle (*Ø_mmax_*,, *Ø_mmin_*) legs were measured relative to the center of mass using the angle tool in *ImageJ*. For both pairs of legs, at the minimum angle (*Ø_hmin_*,, *Ø_mmin_*), the point at which the legs completed the power stroke, was 0°. This means that upon the completion of the cycle, the legs were parallel to the longitudinal, *y* axis, of the beetle. The maximum angles relative to the center of mass during beating, however, were 79.5±10.32° for the hind legs (*Ø_hmax_*) and 120.4±8.14° for the middle legs (*Ø_mmax_*). Previous studies have determined that the acting point of drag on the leg, essentially the position along the length of the leg where 50% of the torque is generated, occurred at a point approximately 68% from the point of attachment for the leg [26]. This means that the acting point of force along the middle legs was 1.41 mm, and 1.81 mm for the hind legs based on the leg measurements. Due to the error that could occur in manually tracing this position, a relationship was established to allow for a more precise calculation of the distance between the acting point of force on the leg and the center of mass. First, it was necessary to determine the position of attachment of the legs relative to the origin, which was achieved by measuring the distance from the origin to the attachment point of the legs using micrographs of the underside of the beetle (**Figure 1B**). From these micrographs, the point of attachment for the middle legs (*P_m_*) was 0.44 mm anterior to the origin, whereas the point of attachment of the hind legs (*P_h_*) was 0.53 mm posterior to the origin. Next, the distance from the center of mass to the acting point of drag on the legs at *Ø_hmin_* and *Ø_mmin_*, *r_hmin_* and *r_mmin_* as shown in **Figure 2**, could be easily calculated as 2.34 mm and 0.97 mm, respectively. Since the distance from the center of mass to the acting point of drag on the legs at *Ø_hmax_* and *Ø_mmax_*, *r_hmax_* and *r_mmax_*, were not linear, calculation of these variables was achieved by using the triangle formed from these angles, the length of the legs at their acting points of force, and the distance from the attachment point to the center of mass of the body. Using these relationships, the triangle could be solved, giving a value of 1.8 mm for the distance from the center of mass to the acting point of drag on the legs at *Ø_hmax_* (*r_hmax_*) and 1.6 mm for the distance from the center of mass to the acting point of drag on the legs at *Ø_mmax_* (*r_mmax_*). All parameter values obtained from the high-speed video analysis are summarized in **Table 2**.

**Table 2 pcbi-1002792-t002:** Swimming parameters obtained from high-speed video analysis.

***U_p_***	Peak leg speed	0.67 m/s
***U_y_***	Average forward velocity	0.0936±0.0226 m/s
***Ø_hmax_***	The maximum angle between the negative *y* axis and the straight line from the center of mass to the acting point of the drag on hind legs	79.5±10.32°
***Ø_hmin_***	The minimum angle between the negative *y* axis and the straight line from the center of mass to the acting point of the drag on hind legs	0°
***Ø_mmax_***	The maximum angle between the negative *y* axis and the straight line from the center of mass to the acting point of the drag on middle legs	120.4±8.14°
***Ø_mmin_***	The minimum angle between the negative *y* axis and the straight line from the center of mass to the acting point of the drag on middle legs	0°
***r_hmin_***	Distance from the center of mass to the acting point of the drag on hind legs at *Ø_hmin_*	2.34 mm
***r_hmax_***	Distance from the center of mass to the acting point of the drag on hind legs at *Ø_hmax_*	1.8 mm
***r_mmin_***	Distance from the center of mass to the acting point of the drag on middle legs at *Ø_mmin_*	0.97 mm
***r_mmax_***	Distance from the center of mass to the acting point of the drag on middle legs at *Ø_mmax_*	1.6 mm

### Calculation of static buoyancy and curvature forces at the air-water interface

Weight support plays an important role in diving and swimming of whirligig beetles. Since whirligig beetles have a density greater than water, the body weight must be supported by the combination of the buoyancy force (*F_b_*) and the curvature force (*F_c_*), i.e, *Mg* = *F_b_*+*F_c_*, where *M* is the mass of the whirligig beetle, and *g* is the gravitational constant. The buoyancy force can be calculated by integrating the hydrostatic pressure over the effective body surface area *S_b_* in contact with the water, and is equal to the weight of fluid displaced above the body and inside the contact line *C*, as shown in **Figure 2**. From this definition, *F_b_* = *ρgV_b_*, where *ρ* is the density of water at 20°C, 998.2 kg/m^3^, *g* is the gravitational constant, 9.8 m/s^2^, and *V_b_* is the volume of water displaced by the body. *V_b_* is equal to the total submerged volume of the body, or *S_b_h*. Using the measurements obtained from the experiments, the buoyancy force on the beetles was 52 µN. Similarly, the weight of the beetles (*Mg*) was calculated as 98 µN. From the relationship outlined above, the curvature force due to the surface tension of water, F_c_, can be calculated as 46 µN and contributes 46.9% of the total weight support. The percentage of weight supported by the curvature force is much lower than water walking insects due to the submersion of the ventral portion of the beetle, whereas a much smaller total volume is submerged in water walking insects. In fact, in an analysis of the weight distribution of a variety of water striders, >90% of the weight was supported by the curvature force in the majority of species tested [28].

### Calculation of Reynold's number

In general, the motion of the legs of aquatic insects is characterized by a high Reynolds number (*Re*), *Re*>>1. Using the equation *Re* = *Uw*/*υ*, where *U* is the peak speed of the object, *w* is the characteristic leg width, and *υ* is the kinematic viscosity of water, which is 9.79×10^−7^ m^2^/s [7], . Based on the experimental measurements obtained from this study, the *Re* of the hind legs was 444.8 and the *Re* of the body was 2042.9. Since the *Re* of both the hind legs and body were much greater than 1, the inertial forces dominate the flow, allowing us to neglect viscous forces when modeling the dynamics of the beetle.

### Experimental analysis of the diving process

**Figure 5:**
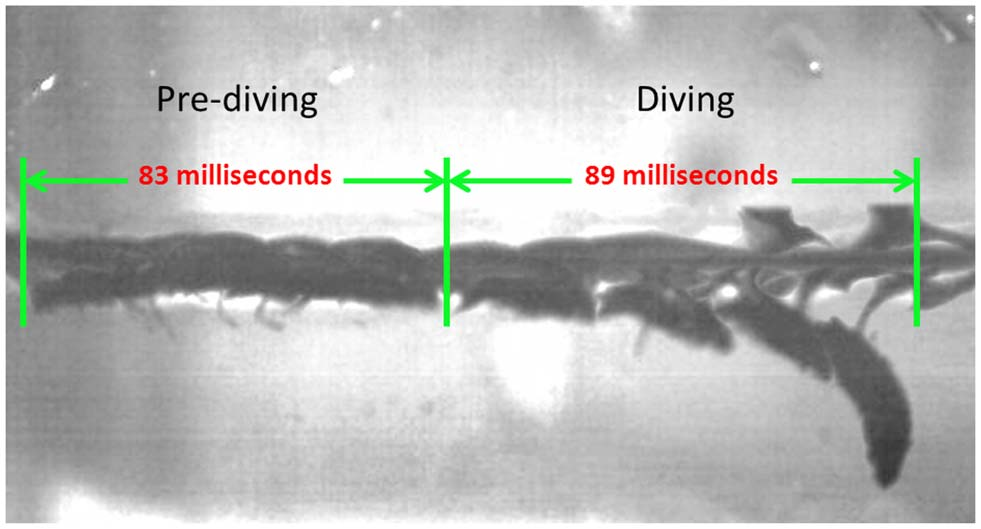
Time-lapse images of the diving process. This image shows the complete diving process, from the 83 ms pre-diving to the 89 ms diving process. To illustrate the diving motion, images captured every 17 ms are overlaid onto each other to show the complete diving motion.

Similar to the approach taken to study the swimming kinematics of the whirligig beetle on the surface of water, analysis of the diving process of whirligig beetles was conducted using a high-speed video camera. **Video S2** shows a typical diving motion from which the parameters related to the diving were obtained. The complete diving process was further divided into a pre-diving stage and a diving stage. The pre-diving stage occurred over the first few leg beats, and was characterized by an oscillation in tilt angle. For the diving stage, the tilt angle constantly increased from the maximum observed in the pre-diving stage. The tilt angle (*γ*) was defined as the degree of body rotation, where a negative value indicates that the head of the beetle dips toward the water, and a positive value indicates that the beetle's head is raised above the water surface. The maximum change in tilt angle observed during the pre-diving process (*γ_max_*) was 10.2°, which can be defined by the difference in the instantaneous maximum and minimum tilt angles produced during one oscillation. After achieving this maximum oscillatory change in tilt angle, the average tilt angle over the remaining oscillations steadily decreased as the beetle further dove, serving as the signal for the initiation of the diving process. During the pre-diving process, the average velocity (*U_pre_*) was relatively slow, 0.1 m/s. This average pre-diving speed was generated by four leg beats, leading to a leg beating frequency (*f_pre_*) of 52 Hz. From the direct observation of the beating motion of the legs in both the pre-diving and diving motion, we found that the legs beat primarily in the *y*–*z* plane, as opposed to beating primarily in the *x*–*y* plane, which was a characteristic of swimming. In other words, during the swimming process, the legs beat more along the side of the body, whereas in diving, the legs beat further underneath the body. Not surprisingly, this change in beating direction leads to a reduced sweep range and a slower velocity, while also providing the force necessary for angular rotation around the x axis. This slower speed, combined with an oscillating *γ*, also allows for a larger wave resistance leading to the formation of a large wave in front of the beetle [1], which will further increase the clockwise rotation by applying a downward force on the anterior portion of the beetle's body. The maximum angular velocity measured in the pre-diving process (*ω_pre_*) was 1090°/s. Upon completion of the pre-diving process, the beetle rapidly dove underneath the water surface by increasing its average leg beating frequency (*f_diving_*) to 100 Hz, and realized a maximum leg beating frequency (*f_max_*) of 142 Hz. This represented a 1.92 fold increase in average leg beating frequency from the pre-diving to diving stage. The maximum velocity (*U_max_*) of 0.56 m/s, was attained during the first leg beat in the diving process, while the average velocity during the diving process (*U_diving_*) was much lower, 0.17 m/s, due to the decrease in velocity associated with increasing fluid resistance. In total, 9 full leg beating cycles were completed during the 89 millisecond diving process, compared to 4 full leg beating cycles over 83 ms for the pre-diving process, as shown in **Figure 5**.

The final set of parameters that could be obtained from the high-speed video of the diving process, were the parameters related to the motion of the legs. Similar to the analysis conducted from the swimming videos, it was necessary to obtain the maximum angle (*Ø_max_*) and minimum angle (*Ø_min_*) between the negative *y* axis and the straight line from the center of mass to the acting point of drag on the legs. In essence, this will be a measure of the sweep range of the legs. Whereas this angle was calculated in the *x*–*y* plane for swimming, as discussed earlier, the motion of the legs during the diving process necessitate the measurement of these angles in the *y*–*z* plane. Using the same procedure described for swimming, the average maximum angle of the hind legs over all leg beats used in diving (*Ø_hmax_*) was found to be 93.9±10.37°, while the minimum angle (*Ø_hmin_*) was 26.1±6.94°. This means that the average sweep of the middle legs during the diving process was 61.8° in the *y*–*z* plane. Similarly, the maximum angle for the middle legs (*Ø_mmax_*) was 124.9±12.7°, and the minimum angle (*Ø_mmin_*) was 43±5.26°. Again, the sweep angle was relative to the origin, center of mass, and thus not the sweep angle from the attachment point of the leg to the body. Using the known distance from the attachment point of the leg to the origin, the distance from the attachment point of the leg to the acting point of the force on the leg, and the angle of the straight line formed from the origin to the acting point of force on the leg, it was possible to solve for the distance between the distance from the center of mass to the acting point of drag on the legs (*r*), as shown in **Figure 2B–C**. It was assumed that the acting point of drag on the legs was 1.41 mm for the middle legs, and 1.81 mm for the hind legs based on the values calculated for swimming. Despite the change in the plane of beating from occurring primarily in *x*–*y* during swimming to primarily in *y*–*z* during diving, this acting point of drag was assumed to remain the same, since the maximum area of both pairs of legs was beating in the *y*–*z* plane, since the maximum area of the legs in both planes are assumed to remain equal with the only change being the angle at which the legs strike and not the orientation of the legs as they generate forward propulsion. The point of attachment of both the hind legs and middle legs to the origin was the same as measured for swimming, 0.53 mm posterior for the hind legs and 0.44 mm anterior for the middle legs. Using these known values, the distance from the center of mass to the acting point of drag on the legs at the maximum and minimum angle, *Ø_max_* and *Ø_min_*, was calculated as, 2.3 mm for *r_hmin_*, 1.7 mm for *r_hmax_*, 1.1 mm for *r_mmin_*, and 1.6 mm for *r_mmax_*. The diving parameters obtained from the experimental analysis of the high-speed capture of the diving process are summarized in **Table 3**.

**Table 3:** Diving parameters obtained from high-speed video analysis.

***U_pre_***	Average pre-diving velocity	0.1 m/s
***U_diving_***	Average diving velocity	0.17 m/s
***U_max_***	Maximum velocity	0.56 m/s
***f_pre_***	Average pre-diving leg beating frequency	52 Hz
***f_diving_***	Average leg beating frequency	100 Hz
***f_max_***	Maximum leg beating frequency	142 Hz
***Ø_hmax_***	The maximum angle between the negative *y* axis and the straight line from the center of mass to the acting point of the drag on hind legs	93.9±10.37°
***Ø_hmin_***	The minimum angle between the negative *y* axis and the straight line from the center of mass to the acting point of the drag on hind legs	26.1±6.94°
***Ø_mmax_***	The maximum angle between the negative *y* axis and the straight line from the center of mass to the acting point of the drag on middle legs	124.9±12.7°
***Ø_mmin_***	The minimum angle between the negative *y* axis and the straight line from the center of mass to the acting point of the drag on middle legs	43±5.26°
***r_hmin_***	Distance from the center of mass to the acting point of the drag on hind legs at *Ø_hmin_*	2.3 mm
***r_hmax_***	Distance from the center of mass to the acting point of the drag on hind legs at *Ø_hmax_*	1.7 mm
***r_mmin_***	Distance from the center of mass to the acting point of the drag on hind legs at *Ø_mmin_*	1.1 mm
***r_mmax_***	Distance from the center of mass to the acting point of the drag on hind legs at *Ø_mmax_*	1.6 mm
***γ_max_***	Maximum pre-diving tilt angle	10.2°
***v_h_***	Velocity of hind leg beat	0.18 m/s
***v_m_***	Velocity of middle leg beat	0.14 m/s
**γ_i_**	Initial tilt angle of the body at the beginning of diving process	−7°
**ω_i_**	Initial angular velocity at the beginning of diving process	−333°/s
***ω_pre_***	Maximum pre-diving angular velocity	1090°/s

### Analysis of simulation results

In order to analyze the swimming and diving processes, simulations were conducted based on the models described above, and the parameters obtained from the experimental analysis listed in **Tables 1**, **2**, and **3**. Two types of trajectories were analyzed from the swimming simulations: trajectories that displayed a net forward motion and trajectories that generated a repeating circular shape. These trajectories were chosen since they were the most relevant, based on the typical swimming patterns observed in nature. Similarly, these types of trajectories were the most important for inspiration of robotics principles, as they directly relate to steering and forward propulsion. For simplification, the leg beating patterns used in the swimming simulations are annotated as follows: *m* or *h* indicates the beating of the middle or hind leg, and the subscript *r* or *l* indicates either the right or left leg. Further, if the legs beat simultaneously, then the notation will be a summation. For example, if the right and left middle legs beat in unison, the notation would be (*m_r_*+*m_l_*). If the leg beats are followed by one another, then there will be a “,” separating the beats. For the case of a middle right leg beat followed by a hind right leg beat, the notation will be (*m_r_*, *h_r_*). For all swimming simulations, the duration of the simulation was 2 seconds, and the initial values for velocity (*U_x_*, *U_y_*), angular velocity (ω), and the turning angle of the body (*β*) were all set to zero.

### Analysis of net forward trajectories

**Figure 6:**
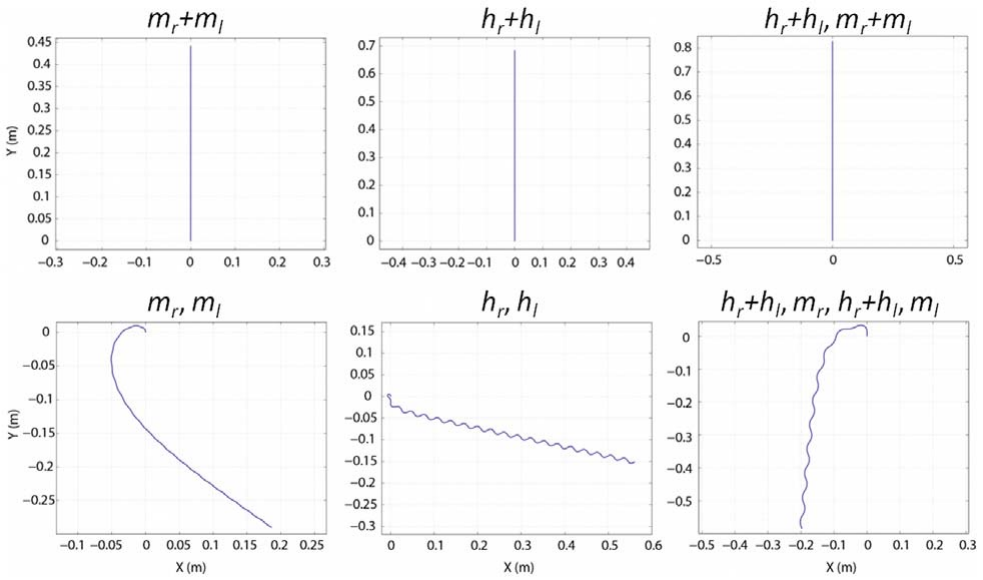
Net forward trajectories from swimming simulations. As indicated above each frame, the beating patterns that generated a true linear path were those where the right and left middle legs (*m_r_*+*m_l_*), hind legs (*h_r_*+*h_l_*), and hind followed by middle legs (*h_r_*+*h_l_*, *m_r_*+*m_l_*) beat simultaneously. In these three cases, the total distance traveled was equal to Δy. For the other three cases where the middle right and left legs (*m_r_*, *m_l_*) and the hind right and left legs (*h_r_*, *h_l_*) beat alternately, and the simultaneous beating of the hind legs followed by the beating of a middle leg (*h_r_*+*h_l_*, *m_r_*, *h_r_*+*h_l_*, *m_l_*) the net forward distance traveled was calculated from a line between the start and end point. Numerical analysis of these trajectories is shown in **Table 4**.

Out of all simulations conducted, six beating patterns that generated a net forward motion were analyzed. Most trajectories simulated showed a high degree of repetitive motions that led to complex patterns, or no net forward motion. The six forward beating patterns analyzed were (*m_r_+m_l_*), (*h_r_+h_l_*), (*h_r_+h_l_, m_r_+m_l_*), (*m_r_, m_l_*), (*h_r_, h_l_*), and (*h_r_+h_l_, m_r_, h_r_+h_l_, m_l_*), as indicated using the notation described above. The trajectories for these motions are shown in **Figure 6** and the values calculated from these trajectories are shown in **Table 4**. Based on the results obtained from these simulations, the highest forward velocity, 0.5268 m/s, was achieved for (*h_r_+h_l_, m_r_+m_l_*), the simultaneous beating of the hind legs followed by the simultaneous beating of the middle legs, while the lowest maximum speed, 0.2473 m/s, was attained for (*m_r_, m_l_*), the alternate beating of the middle legs. Similarly, the greatest net forward motion, 0.8284 m, was observed for (*h_r_+h_l_, m_r_+m_l_*) and the least, 0.3445 m, was observed for (*m_r_, m_l_*). However, if we consider these beating motions in terms of efficiency, then the most efficient strategy would be the one that results in the largest net forward motion per leg beat. From a biological perspective, the energy expenditure relative to distance traveled is important, since excessive leg beating will lead to exhaustion. Using this definition of efficiency, the most efficient strategy of the Whirligig beetle was calculated to be (*h_r_+h_l_*), the simultaneous beating of the hind legs, at 17.1 mm/beat, followed by (*h_r_, h_l_*), the alternate beating of the hind legs, at 14.5 mm/beat. What can clearly be concluded from this efficiency value is the importance of the hind legs in forward propulsion. When comparing (*h_r_+h_l_*) to (*m_r_+m_l_*), the average speed and net forward distance traveled using the hind legs is 1.55 times larger. In terms of the strategy that was the most efficient per beat in total distance traveled, (*h_r_, h_l_*), 17.7 mm/beat, was the most efficient followed by (*h_r_+h_l_*), 17.1 mm/beat. In general, over the total distance traveled, strategies using the hind legs only (*h_r_+h_l_*) were 1.55 times more efficient than those with the middle legs only (*m_r_+m_l_*). When considering the total distance traveled per beat as a measure of efficiency, (*h_r_+h_l_, m_r_+m_l_*) was the worst strategy 10.3 mm/beat, despite moving the greatest overall distance. It should be noted that this pattern of beating was not observed in the experimental study, and may be the result of the low efficiency of this beating pattern.

**Table 4:** Analysis of forward trajectories.

Beating Pattern	Maximum forward speed (m/s)	Total number of leg beats (Ct)	Linear distance from start to finish (m)	Total distance traveled (m)	Average Speed (m/s)	Linear distance/beat (m/beat)	Distance Traveled per beat (m/beat)
*m_r_+m_l_*	0.2809	40	0.4423	0.4423	0.22115	0.0110	0.0110
*h_r_+h_l_*	0.4659	40	0.6853	0.6853	0.34265	0.0171	0.0171
*h_r_+h_l_, m_r_+m_l_*	0.5268	80	0.8284	0.8284	0.4142	0.0104	0.0103
*m_r,_ m_l_*	0.2473	40	0.3445	0.4431	0.22155	0.0086	0.0111
*h_r_, h_l_*	0.4021	40	0.5806	0.7108	0.3554	0.0145	0.0177
*h_r_+h_l_, m_r_, h_r_+h_l_, m_l_*	0.5000	60	0.6169	0.7622	0.3811	0.0103	0.0127

The results obtained from the simulations can be interpreted from a biological perspective to understand reasons of the beating patterns, and trajectories observed in nature. One of the hallmarks of the movement of whirligig beetles is their overall rounded trajectories, and the common observation of *S*-shaped trajectories. Based on the analysis from the simulations, *S*-shaped trajectories generated by (*h_r_, h_l_*) represent the most energetically favorable strategy for covering a large distance with an efficiency of 17.7 mm/leg beat (**Table 4**). While this may seem counterintuitive, the anatomical structure of the beetle may dictate the efficiency of this strategy over a linear trajectory. In terms of predator avoidance and escape, an effective “flight” response would allow the beetle to rapidly move away from the perceived threat. In addition, to maximize the distance between the beetle and the threat, a strategy must be chosen that would balance the energetic costs of escape, in terms of both speed and distance traveled. A short beating duration using (*h_r_+h_l_, m_r_+m_l_*) would result in a maximum burst speed, but would most likely lead to a tiring of the beetle due to the higher energetic cost. This may explain why linear trajectories have rarely been observed when studying the whirligig beetles. The most efficient strategy that would allow the beetle to cover a large distance with minimal energy expenditure would be the S-shaped trajectory generated by the alternate beating of the hind legs. This strategy would allow the beetle to outdistance the predator while moving along a less predictable trajectory, without leading to exhaustion, and may explain why S-shaped trajectories are commonly observed in nature.

Another result from the analysis of the leg patterns used in swimming was the obvious propulsive advantage of using the hind legs over the middle legs. This may explain why several researchers have noted that the hind legs are often observed beating twice as fast as the middle legs [14]. The results from this study suggest that, rather than a physiological constraint that allows the hind legs to beat twice as fast, the hind legs beat twice as much due to the benefit in overall propulsion efficiency. The middle legs would then be expected to contribute to stability control to maintain a given path and prevent a loss of control during swimming. This will be analyzed in greater detail in the following section.

### Analysis of the circling trajectories

**Figure 7:**
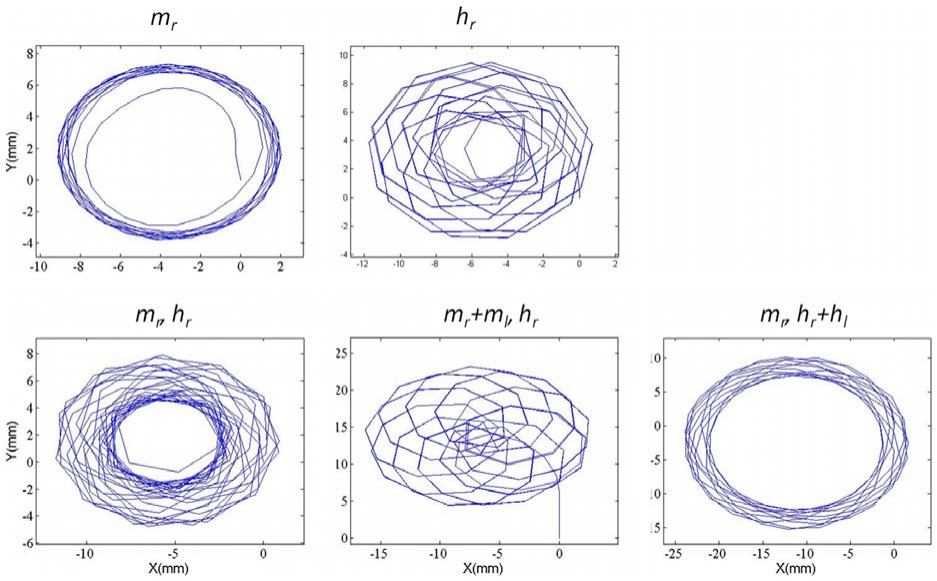
Circling trajectories from swimming simulations. The circular trajectories obtained from the swimming simulations are illustrated above. Based on the simulations, only three beating patterns stabilized to form a consistent circular trajectory, the middle right leg only (*m_r_*), the middle right followed by the hind right (*m_r_*, *h_r_*), and the middle right followed by the simultaneous beating of the hind legs (*m_r_*, *h_r_*+*h_l_*). The other beating patterns analyzed produced unstable trajectories, resulting in trajectories not observed in nature. Numerical analysis of the circular swimming trajectories is shown in **Table 5**.

Five of the simulations displayed an overall circular trajectory as shown in **Figure 7**. These beating patterns demonstrating an overall circular trajectory were (*m_r_*), (*h_r_*), (*m_r_, h_r_*), (*m_r_+m_l_, h_r_*), and (*m_r_, h_r_+h_l_*), and the values associated with their analysis are shown in **Table 5**. From these trajectories, we can conclude that the most efficient, in terms of total distance traveled per leg beat was (*h_r_*), 30.9 mm/beat, which was also the most efficient out of all the leg beating patterns analyzed in this study. In fact, this was 1.74 times more efficient than the most efficient beating pattern observed from the net forward trajectories (*h_r_*, *h_l_*) of the beetle. Comparison of the efficiency of the motion generated by patterns (*h_r_*) and (*m_r_*), the use of only one hind leg or middle leg, led to a 1.74 fold increase in the overall distance traveled per leg beat, similar to the results observed from the forward trajectories. As expected, motion generated by (*h_r_*) had a much higher average angular velocity, 4336.52°/s, compared to (*m_r_*), 1958.74°/s. This is a 121% fold increase from the hind leg only to middle leg only beating, which is likely due to the attachment point of the rear legs being 0.53 mm posterior to the origin of the body. In addition, the longer length of the hind legs and their larger propulsive area, further have a significant impact on turning. The beating pattern that had the greatest average angular velocity, 4742.54°/s, was the middle right leg followed by the hind right leg (*m_r_, h_r_*). This was in accordance with several studies that have pointed out that the most common leg beating pattern in the circling behavior of whirligig beetles is the beating of the outboard legs, which in the case of the simulation was (*m_r_, h_r_*). Similarly, the values for angular velocity obtained from the simulations were very close to the value of 4428°/s obtained in previous studies. The beating patterns that were most likely to reproduce a truly circular trajectory, were (*m_r_+m_l_, h_r_*) and (*m_r_, h_r_+h_l_*), both with nearly the same efficiency, distance traveled, and number of beats. There was however a significant difference in the average angular velocity among these beating patterns, where (*m_r_+m_l_, h_r_*) was 2.15 times faster than (*m_r_, h_r_+h_l_*), resulting in a difference in the radius of curvature.

**Table 5:** Analysis of circling trajectories.

Beating Pattern	Maximum forward speed (m/s)	Average angular velocity (°/s)	Total number of leg beats (Ct)	Total distance traveled (m)	Average Speed (m/s)	Distance Traveled per beat (m/beat)
*m_r_*	0.2108	1958.74	20	0.3547	0.1774	0.0177
*h_r_*	0.3903	4336.52	20	0.6188	0.3094	0.0309
*m_r_, h_r_*	0.4208	4742.54	40	0.7134	0.3567	0.0178
*m_r_+m_l_, h_r_*	0.4231	4218.57	60	0.7634	0.3817	0.0127
*m_r_, h_r_+h_l_*	0.5099	1958.74	60	0.7709	0.3854	0.0128

From the analysis of the circling trajectories, we see again that the hind legs were more effective per leg beat, and also were able to produce a larger angular velocity when compared to the middle legs. Unlike the forward trajectories, however, as shown in **Figure 7**, the trajectory from only the right hind leg beating, (*h_r_*), was more chaotic due to the larger increase in the angular velocity. The most common trends observed in nature were the circling trajectories produced by (*m_r_+m_l_, h_r_*), the simultaneous beating of the middle legs followed by the beating of the right hind leg, and (*m_r_, h_r_+h_l_*), a middle right leg beat followed by the simultaneous beating of the hind legs. By using the middle legs to balance out the large angular velocity of the hind legs, it is possible to get a much more stable path. The exact reason that the beetles prefer rapid circular trajectories remains unknown; however, from this study, it can be concluded that these patterns appear to be more energy efficient than other motions. Biologically, the energy conserved by using these motions may dictate their use for both predator avoidance, and prey capture.

To determine the effect of errors associated with the measurement of the swimming parameters obtained experimentally, parametric analysis was conducted to examine how perturbation of these terms affected the swimming simulations. In order to maintain realistic variation among these terms, they were perturbed by the percent change associated with the standard deviations reported in **Table 1**. The complete results from this parametric analysis are included in the **Text S1**.

### Analysis of diving simulation

**Figure 8:**
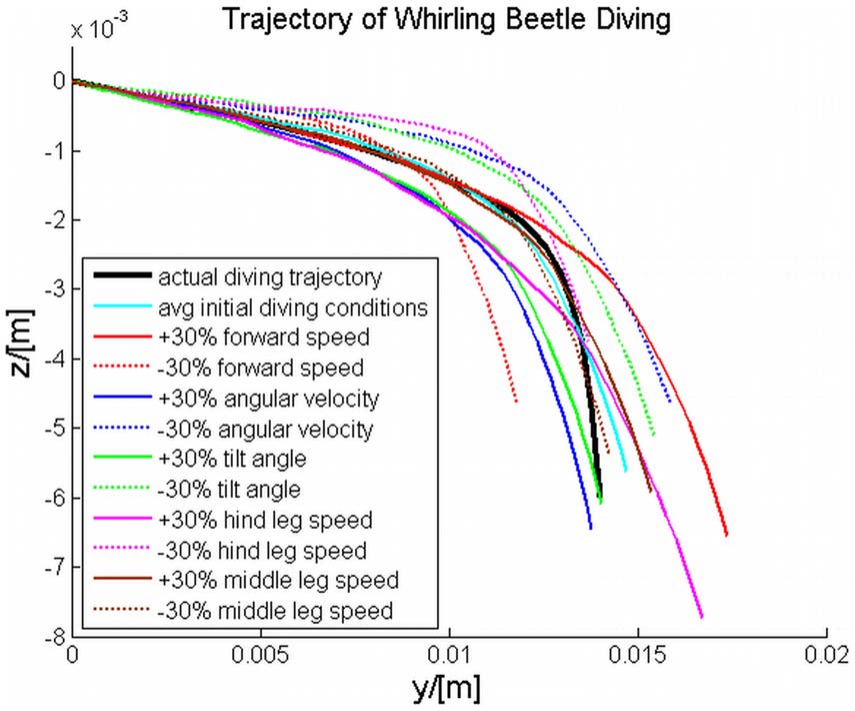
Results from the diving simulations. Simulation results of diving with different initial conditions. The initial values of forward speed (0.17 m/s), angular velocity of the body (−333°/s), tilt angle of the body (7°), striking speed of the hind legs (0.18 m/s), and striking speed of the middle legs (0.14 m/s), were varied ±30% to determine the effect on the diving trajectory. Each of these terms was varied ±30%, with the other terms held constant, to determine their effects on the overall trajectory. The values generated the closest diving trajectory as observed in the experimental studies was with an initial speed of 0.17 m/s, angular velocity of −333°/s, tilt angle of −7°, hind leg speed of 0.18 m/s, and middle leg speed of 0.14 m/s.

A numerical study of the diving process was also conducted to validate the dynamics model for the diving process. The values of morphological parameters were given in **Table 1**. The parameters obtained for diving are listed in **Table 3**. The initial values of the forward velocity, the angular velocity, and the turning angle of the body were chosen to be 0.17 m/s, −333°/s, and −7°, which were consistent with the parameter values obtained at the start of the diving process, based on the experimental study. The initial depth of the body under the free surface of water was set to be 0.74 mm, with the assumption that the center of mass was in the plane of free surface at *t* = 0. The 89 ms diving process was simulated, as shown in **Figure 5**, using the body coordinate system. For diving, the hind legs beat together with a speed of 0.18 m/s, followed by simultaneous middle leg striking at 0.14 m/s. The leg beating pattern that was observed in the diving video, and used in the simulation, contained 6 hind legs beats and 3 middle legs beats. Additionally, the timing of the leg beats used for the simulation was the same as that observed in the diving video. Despite careful analysis of the diving videos, determination of the dynamic changes in the buoyancy and curvature force during diving proved difficult. Since these forces could not be obtained experimentally, it was necessary to establish a coefficient to explain the dynamic changes in these forces. As such, the combination of the buoyancy and curvature force was estimated to change nonlinearly according to the segmented function 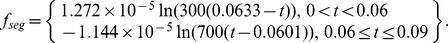 This segmented function was established to explain the slow change in tilt angle over the first 60 ms, followed by the rapid change in tilt angle during the final 29 ms, as shown in **Figure 5**. The change in tilt angle will have a large impact on the value for the curvature force term, since the contact line length will change correspondingly, and the buoyancy force will more gradually increase to a maximum when the beetle is completely submerged. Using this approximation as the change in both curvature and buoyancy forces, a simulation result was attained that closely followed the actual diving trajectory observed in the experimental study, **Figure 8**. This simulation used the initial values from the experimental study, as indicated above. To determine if any of these parameters had a crucial effect on the beetle's ability to dive, further simulations were conducted by varying the initial values of the forward velocity, angular velocity, turning angle of the body, hind leg speed, and middle leg speed.

The initial conditions of forward velocity, angular velocity, tilt angle of the body, hind leg speed, and middle leg speed were 0.17 m/s, −333°/s, −7°, 0.18 m/s, and 0.14 m/s, respectively. For each parameter, the value was varied ±30% of the initial value, while the other values were kept constant. A 30% increase in any of the following velocity terms, forward velocity, hind leg speed, and middle leg speed, led to an increase in the distance traveled in the y-axis and the depth of the dive in the *z*-axis. Of these parameters, the middle leg speed had the smallest effect on the overall diving trajectory. This was due to the leg beating pattern modeled, where only 3 middle leg beats occurred, compared to 6 for the hind legs. The lower propulsive area of the middle legs also led to only a minimal change in the observed diving trajectories for ±30%. The parameter that had the largest effect on the diving trajectory was the hind leg speed, since these legs have a large effect on propulsion, and there are twice as many beats in the diving simulation. For the larger hind leg speed (the solid magenta line), the beetle will dive farther in y 13.57% and deeper 37.6%, compared to the simulated initial trajectory. Unlike the previous velocity terms, increases in the angular terms, tilt angle and angular velocity, led to diving trajectories with decreased distance traveled in y, 4.53% and 6.37%, respectively. However, increases in these terms still led to an increase in the depth of the dive. Biologically this can be explained, since the increase in the angular terms equates to the beetle more rapidly entering the water, leading to a deeper dive with a decrease in the length of the dive (distance in y). Overall, the simulation data showed that changes in any of the initial terms used for the simulation played only a minor role compared to dynamic changes in the buoyancy and curvature forces. With 2D data, it was not possible to determine the absolute dynamic changes in these terms, thus, a 3D setup must be used to obtain experimental values for these forces.

### Bio-inspiration for robotic design

As discussed above, the high speed of swimming and diving, large angular velocity of the body in swimming, and unique strategy for maximizing the effective area during a propulsive stroke, are key features for bio-inspiration of robot design. Based on the simulations, we were able to determine that with the morphology present in the whirligig beetles, it was energetically more efficient to use an alternating beating of the hind legs, even though this beating pattern does not move in a straight line. This is consistent with experimental observations of curved trajectories being the most common and can be used for potential propulsion system design in small swimming robots under similar conditions. The *S*-shaped swimming motion, commonly observed in the wild, thus represents a more efficient strategy than linear motion, assuming a robot with the same size and morphology as the beetle. This is a very interesting phenomenon and could be used for small swimming robot path planning to improve energy efficiency. Similarly, the attachment point of the legs relative to the origin, allows the beetle to attain an incredible angular velocity by paddling the outboard legs in an alternating fashion. Another important source of inspiration for robotic design is the morphology of the hind leg, which is crucial in both propulsion and turning. By minimizing the drag in the recovery stroke, and maximizing the effective propulsive area in the power stroke, the whirligig beetle is able to achieve rapid speeds with a highly efficient motion. By designing an oar, with similar morphology to the hind legs of the whirligig beetle, it would be expected that the swimming device would achieve much more efficient propulsion. Many studies have sought to develop biomimetic robots to achieve a goal similar to that of their biological counterparts. These robots include water-walking robots based on the water strider [29], [30], [31], snake inspired robots [32], [33], and even wall climbing gecko robots [34], [35]. Based on the highly efficient design of the whirligig beetle's legs, we envision a bio-inspired robot that can mimic the design of both the hind and middle legs of the beetle.

Similarly, by using comparable body geometry, the robot would be able to achieve a high angular velocity by beating the rear paddles, while adjusting its trajectory, in effect, steering with the middle paddles. In addition, the motion of the legs during both swimming and diving provides a source of inspiration. In swimming, the legs beat predominantly in the *x*–*y* plane, whereas in diving, the legs beat predominantly in the *y*–*z* plane. By changing the plane of the beating motion, the beetle is able to achieve an angular rotation in the *y*–*z* plane relative to the surface of the water, creating an angle that will allow it to break the surface tension of the water and dive. This seemingly subtle change has potential applications in robot design. For example, by designing a robot with similar leg structures, the robot would be able to swim or dive using the same propulsive structures, which are dependent on the plane of beating. The ability to adjust this angle of the leg beating plane during swimming would also enable a much larger set of trajectories offering more precise and finer control over the desired swimming path. The pattern of the leg beating used by the beetle to alter its angle of the leg beating plane, and eventually break the surface tension, may also represent an ideal pattern that could be implemented into a diving robot.

Yet another source of inspiration comes from the ability of the beetle to maintain an overall ellipsoid shape when the legs are not beating. As shown in **Figures 1** and **3**, the beetles could fold the legs underneath the body, thus reducing drag. In terms of the body, the point of attachment of the legs allows them to swing out away from the body during beating, but to return underneath the body when not beating. This allows the beetle to effectively coast after each beat, which conserves energy. This phenomenon was observed in nearly all beetle studies, where after a beat, there is a period of time where the beetle will decelerate and “coast” prior to initiating another beat. This was also typically observed in turning, where either a right or left leg would beat, and the beetle would continue to rotate without further beating. Previous robotics studies have sought to mimic the morphology of other biological organisms to design more advanced and efficient robots [36], [37], [38]. By designing a swimming robot that can effectively coast and reduce drag when not using its propelling structures, it would be possible to reduce the energetic costs required to move the robot over an equal distance, leading to a more efficient strategy and utilization of its energy.

### Conclusion

By integrating experimental studies and theoretical analysis, this research has made several contributions to the study of dynamics and kinematics involved in the swimming and diving of whirligig beetles with potential applications in bio-inspired robotics. First, it was discovered that the whirligig beetle dives by altering the plane in which the legs are beating, from *x*–*y* in swimming, to *y*–*z* in diving. The dynamics model developed in this study further supports this claim. Second, results from the swimming dynamics model demonstrated that the most efficient strategy for net forward motion was the beating of the hind legs simultaneously (*h_l_*+*h_r_*). However, the most efficient beating over the total distance traveled was observed in S-shaped swimming (*h_r_*, *h_l_*), alternating beating of the hind legs, and circling (*m_r_, h_r_*), alternating beating of the middle right and hind right legs, (*m_r_*), the beating of the middle right leg only, and (*h_r_*), the beating of the hind right leg only. This finding explains why these swimming trajectories are most commonly observed in nature. Third, analysis of the beating patterns used in the generation of circular trajectories showed that the largest average angular velocity was attained by the beating of a middle leg followed by the beating of a hind leg on the outboard side of the turn. This was consistent with the experimental observations of circular swimming seen in the wild, where this was the most common beating pattern. Comparison of simulations using either the hind or middle legs showed that the hind legs were able to generate larger forward propulsion, and also a greater turning angle when compared to the middle legs. This led to the conclusion that the middle legs serve mainly to control the stability of the beetle, and for path correction. This was confirmed by the generation of stable circular trajectories with middle leg beating, compared to unstable trajectories observed as shown **Figure 5** without the presence of the middle legs.

Based on the results obtained from this study several key points of inspiration were identified related to the design of swimming/diving robots. The unique morphology of the legs, allowing for greater increase in area during the power stroke, through the use of collapsible laminae, may lead to the design of more advanced paddles or oars. Next, the ability of the legs to fold underneath the body and maintain an ellipsoidal body shape, reduces the drag on the beetle and allows it to effectively coast, preventing the need for constant beating. Finally, by changing the plane of beating of the legs, an angular rotation can be created that provides the angle necessary for penetration below the surface, essentially diving. By combining these principles, it may be possible to build a more efficient bio-inspired swimming/diving robot.

## Methods

### Experimental procedures

The whirligig beetles were collected from the Tennessee River and maintained in an aquarium at room temperature. A system consisting of several components was assembled to generate a platform for high-contrast imaging of the beating legs. A Powerview HS-650 (TSI, Inc., Shoreview, MN) particle tracking camera with a Sigma 18–200 lens was used to capture the leg beating pattern and swimming motion at more than 800 fps. Using this setup, we were able to track the leg beating pattern throughout its entire movement. Other components of the system included Camware (PCO AG) and ImageJ (NIH), a motion analysis software package. Camware allowed for the playback of videos at different controllable rates. The speed of each leg in both the swimming and diving processes was analyzed by conducting traces of the movement over individual frames using ImageJ. An SEM study was also conducted on the model LEO 1525 from Carl Zeiss equipped with a ‘Gemini’ column.

### Modeling the swimming and diving dynamics

The swimming of whirligig beetles has been well-studied, with all previous studies operating under the assumption that the body is rigid, with no flexibility, and that the legs behave as “rigid paddles” or “swimming blades” during a swimming stroke [1], [2], [4], [5], [8]. While the legs are actually separated into 3 distinct segments (femur, tibia, and tarsi), we define a swimming stroke as starting, when the leg is completely unfolded and extended from underneath the body, and then terminating when the leg begins to fold back underneath the body and returns to its starting position. Using this definition, the angular sweep of a single stroke can be calculated as an arc, as illustrated in [4], and Figure 2 of the manuscript. Using this definition of a stroke is consistent with the previous works [4], [8], the flexibility of the legs during a swimming stoke can be neglected. Similarly, the swimming laminae may exhibit some minor flexion, but for the purposes of this study, this flexibility is negligible.

In this work two models were developed to study the swimming and diving of Whirligig beetles. Both of the models were used to obtain simulations of these processes. The complete description of the models and code are contained in the **Text S1**; however, the key factors involved in the development of the models are identified in this section. In the swimming model, the key hydrodynamic forces involved in the model are the fluid resistive force in x and y (*f_rx_* and *f_ry_*), and the drag force of the legs in x and y (*f_mx_*, *f_my_*, *f_hx_*, *f_hy_*). Since the swimming model is 2D, and the beetle is assumed to always be on the surface of the water, the buoyancy and curvature forces, *F_b_* and *F_c_*, are neglected because they do not oppose the direction of motion. Unlike *F_b_* and *F_c_*, however, wave resistance can significantly affect the motion of the beetle. Previous studies have shown that wave resistance and fluid resistance are of similar magnitude for whirligig beetles, however, all current models for calculating wave resistance assume an absence of contact between the object and the water surface, quantification of wave resistance is not currently possible based on this assumption [1]. Following the model for wave resistance provided by [39], and assuming that the beetle has no size, but a defined weight, the magnitude of wave resistance would be discontinuous producing a maximum value at 23 cm/s, a value of 0 at speeds <23 cm/s, and decrease exponentially at speeds >23 cm/s [1].

Considering that the diving model functions in the y–z plane, as opposed to the x–y plane of the swimming model, the hydrodynamic forces considered in this model differ from those defined in the swimming model. The fluid resistive forces in y and z (*f_ry_* and *f_rz_*), and the drag force of the legs in y and z (*f_my_*, *f_mz_*, *f_hy_*, *f_hz_*) are considered, similar to the swimming model. Again, wave resistance was neglected from the diving model for the same reasons as the swimming model, described above. However, since diving acts in opposition to both buoyancy and curvature forces, these forces must be considered in the diving model. As described above in the analysis of the diving simulations, due to limitations in experimentally determining the dynamic changes in the buoyancy and curvature forces, a segment function (*f_seg_*) was created to account for these forces. This segment function accounted for the slow change in tilt angle over the first 60 ms of the diving process, compared to the rapid change in tilt angle during the final 29 ms of the diving process. The change in tilt angle is related to the curvature force, by changing the contact line length, and thus this approximation was used in simulations of the diving process.

In both models, the forces generated by the creation of vortices from the movement of the legs and body have been neglected due to the use of an experimentally measured drag coefficient. The drag coefficient of the beetle, *C_db_*, used in this study was obtained from [26], where this parameter was calculated for a variety of Whirligig beetles using both wind tunnel and water channel experiments. In these experimental studies, the value for *C_db_* accounts for the vortices created by the beetle. To further confirm that the coefficient of drag accounted for the formation of the resulting vortices, we calculated the force from the body vortices of the Whirligig beetles used in this study as outlined in the equations provided by [40]. The force from the formation of vortices by the body was found to be 18 µN, while our calculation of the drag force using the coefficient of drag at the same velocity was 24 µN. Considering that other factors were involved in the drag calculation used in this study, the values indicate that the force from the formation of vortices has been accounted for in our drag calculation. Further, other studies have used the drag coefficient from [26] to calculate the drag from Whirligig beetles, and obtained values similar to those obtained in this study [1].

## Supporting Information

Figure S1 **The swimming dynamics analysis of whirligig beetles.** (A) Both the rowing of middle legs and striking of hind legs will generate the force component in the longitudinal and lateral directions. The lateral force component will form the centripetal acceleration to change the direction of the forward speed. The net torque produced by the striking of one hind leg will result in a rotation of the whirligig beetle's rigid body. (B) The body coordinate system of the whirligig beetle. The increment of displacement in the *y* direction is assumed to always be positive. The increment of displacement in the *x* direction is negative in the left hand direction. The increment of the turning angle of the body is positive in the counterclockwise direction.
(TIF)

Figure S2 **The dynamics modeling for the diving process of the whirligig beetle.** (A) the pre-diving process, (B) the diving process. The diving is powered by a torque *T_x_* combining the forces generated by striking the middle and hind legs and the fluid resistance. The beetle will turn its body perpendicular to the free water surface, towards decreasing the water resistance and surface tension.
(TIF)

Table S1 **Parametric analysis of swimming motion.** Simulations were conducted to determine the effect of perturbation of the key swimming variables ±10% of the measured values. The effect of the perturbed variables on maximum forward speed and distance traveled per beat were recorded two forward trajectory simulations (*hr+hl*, *mr+ml*) and (*hr+hl*,*mr*,*hr+hl*,*ml*), and one circular trajectory simulation (*mr*,*hr*). In addition, the average angular velocity was determined for the circular trajectory simulation.
(TIF)

Text S1 **Supporting information text.** Included in the supporting text are the details for the parametric analysis of the swimming variables, more detailed methods detailing the model, as well as, the annotated swimming and diving code.
(DOCX)

Video S1 **Real-time swimming of** ***Gyrinidae*** **.** This video showed that the whirligig beetle folded the forelegs under the body, propelled forward using middle legs and hind legs, and turned by beating hind legs in different phases. The video is played back at 30 fps to demonstrate the motion. This video corresponds to the series of figures in **Figure 4**.
(WMV)

Video S2 **Real-time diving of** ***Gyrinidae*** **.** The Video captured the whole diving process including pre-diving, diving, and post-diving, as well as the wave generation. Each frame in the video represents ∼1.7 ms. This video corresponds to the figures in **Figure 5**.
(WMV)
