# Supplementary figures and images for: Experimental Studies and Dynamics Modeling Analysis of the Swimming and Diving of Whirligig Beetles (Coleoptera: Gyrinidae)
Source: PLoS Comput Biol. 2012 Nov 29;8(11):e1002792. doi: 10.1371/journal.pcbi.1002792 (PMC3510063; doi:10.1371/journal.pcbi.1002792)

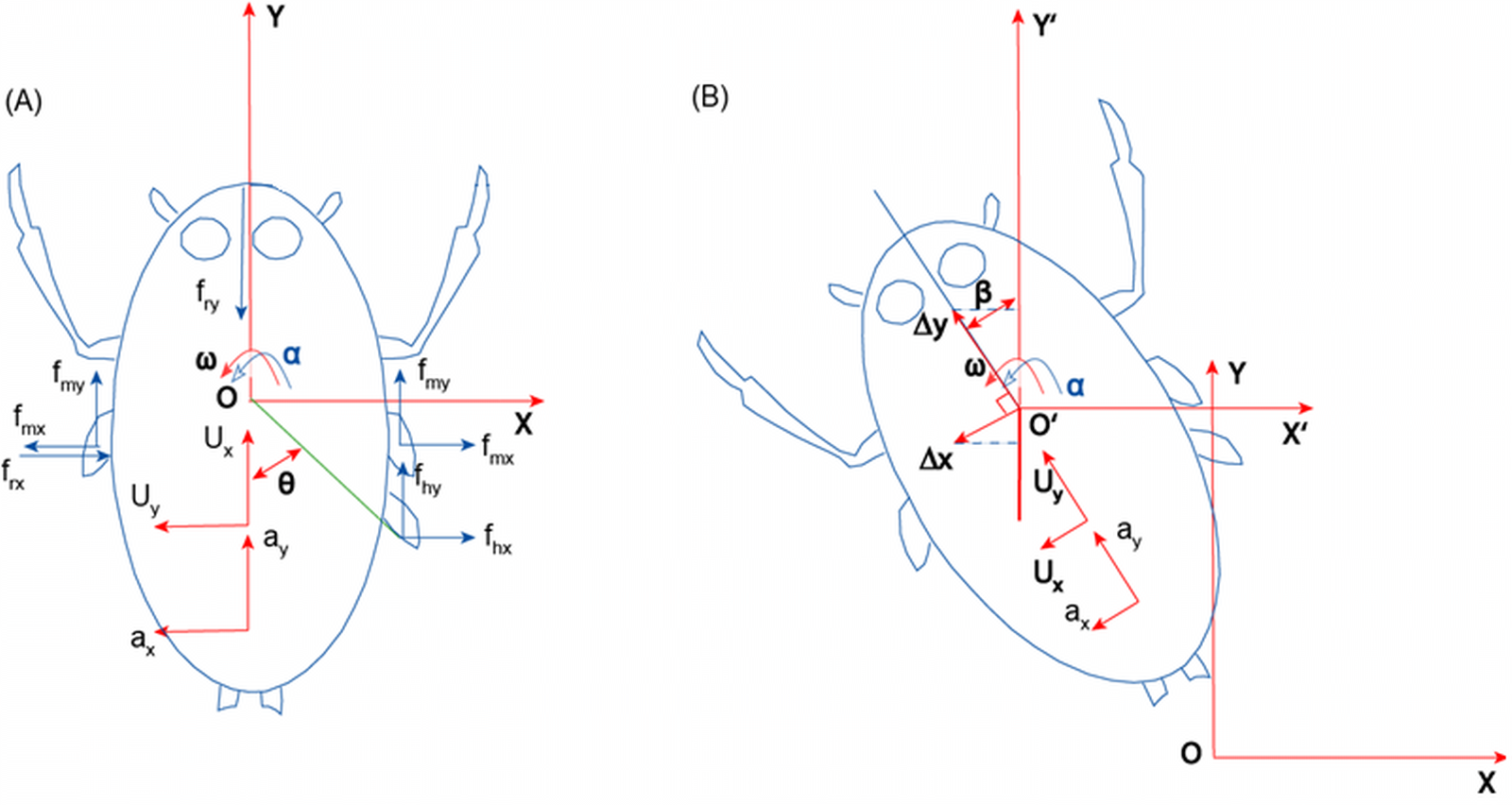

Supplement: Figure S1 — The swimming dynamics analysis of whirligig beetles. (A) Both the rowing of middle legs and striking of hind legs will generate the force component in the longitudinal and lateral directions. The lateral force component will form the centripetal acceleration to change the direction of the forward speed. The net torque produced by the striking of one hind leg will result in a rotation of the whirligig beetle's rigid body. (B) The body coordinate system of the whirligig beetle. The increment of displacement in the y direction is assumed to always be positive. The increment of displacement in the x direction is negative in the left hand direction. The increment of the turning angle of the body is positive in the counterclockwise direction. (TIF) [file pcbi.1002792.s001.tif]

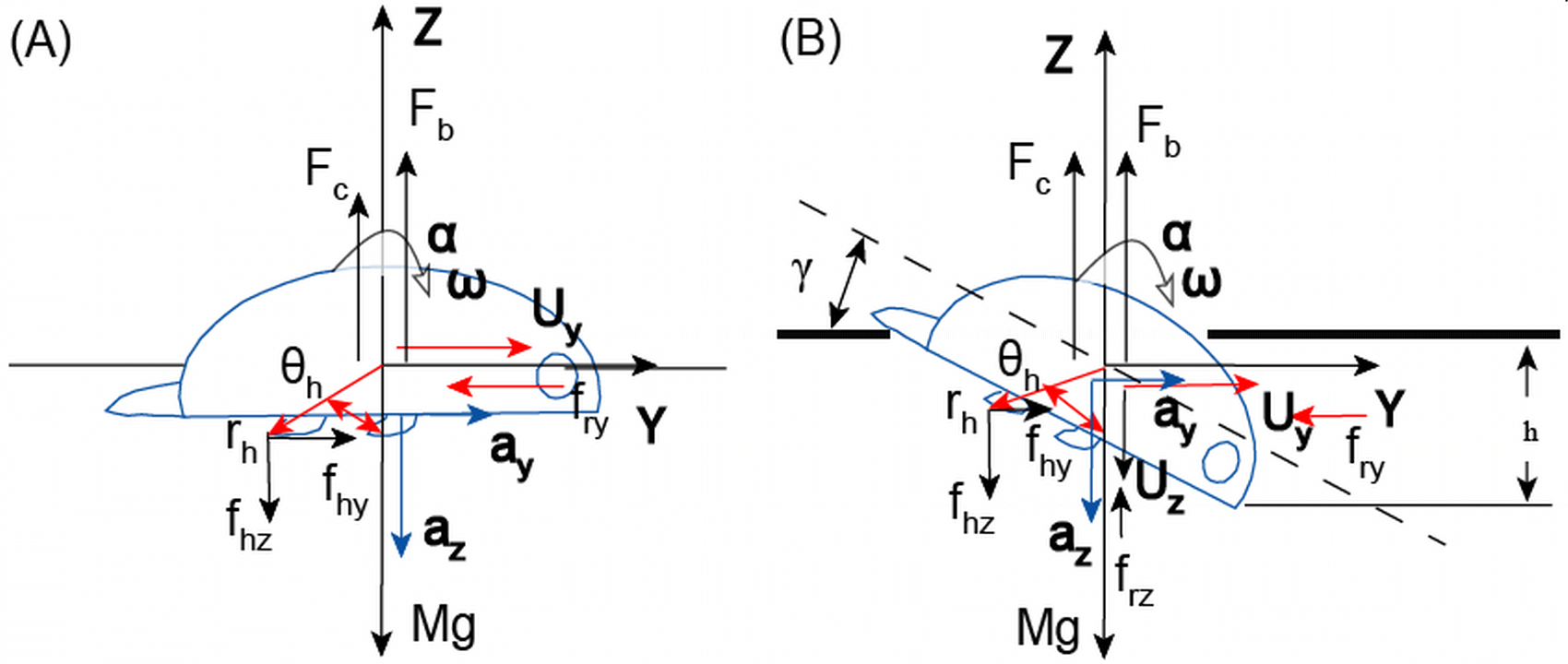

Supplement: Figure S2 — The dynamics modeling for the diving process of the whirligig beetle. (A) the pre-diving process, (B) the diving process. The diving is powered by a torque Tx combining the forces generated by striking the middle and hind legs and the fluid resistance. The beetle will turn its body perpendicular to the free water surface, towards decreasing the water resistance and surface tension. (TIF) [file pcbi.1002792.s002.tif]

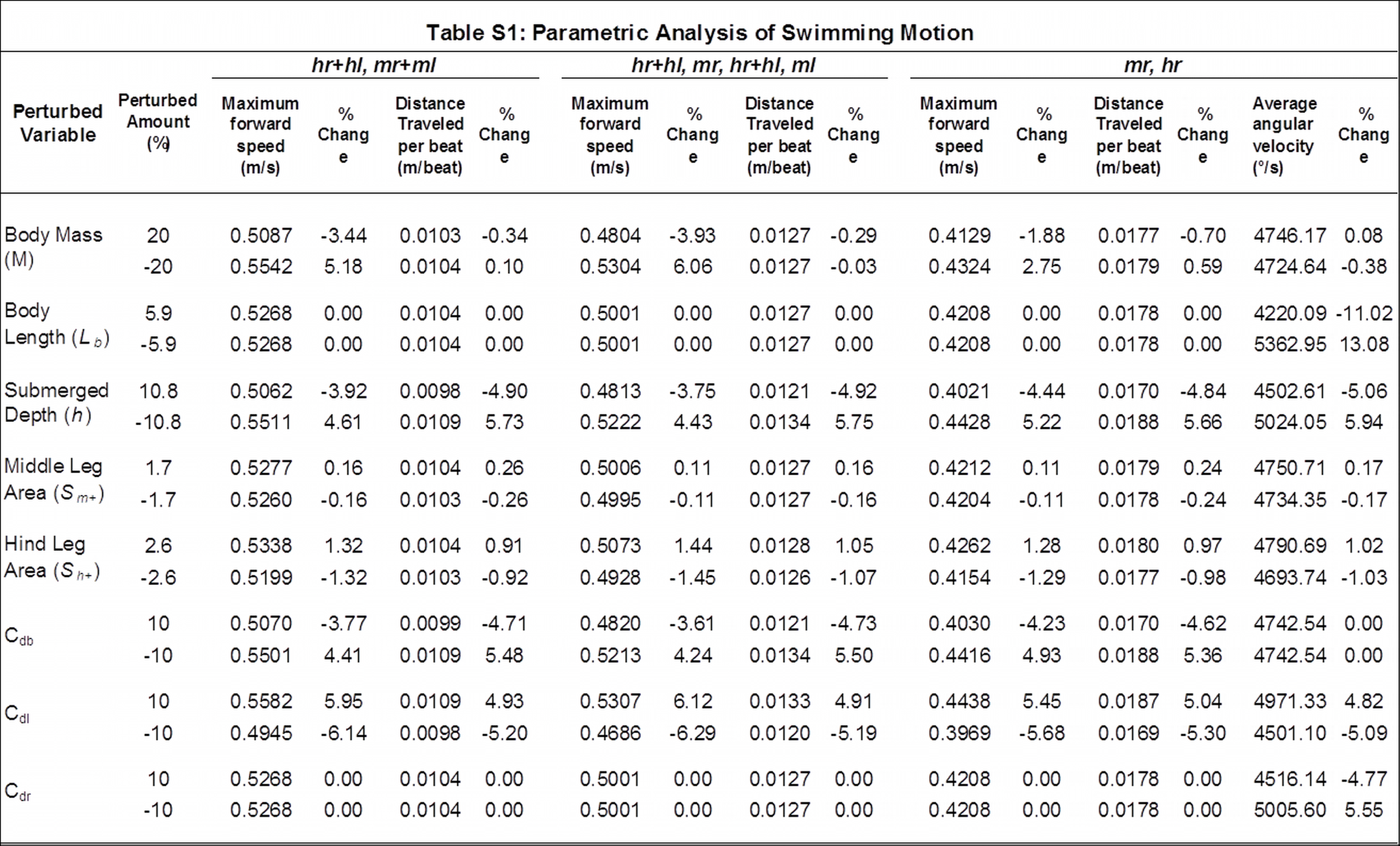

Supplement: Table S1 — Parametric analysis of swimming motion. Simulations were conducted to determine the effect of perturbation of the key swimming variables ±10% of the measured values. The effect of the perturbed variables on maximum forward speed and distance traveled per beat were recorded two forward trajectory simulations (hr+hl, mr+ml) and (hr+hl,mr,hr+hl,ml), and one circular trajectory simulation (mr,hr). In addition, the average angular velocity was determined for the circular trajectory simulation. (TIF) [file pcbi.1002792.s003.tif]
