# Supplementary material for: Experimental Studies and Dynamics Modeling Analysis of the Swimming and Diving of Whirligig Beetles (Coleoptera: Gyrinidae)
Source: PLoS Comput Biol. 2012 Nov 29;8(11):e1002792. doi: 10.1371/journal.pcbi.1002792 (PMC3510063; doi:10.1371/journal.pcbi.1002792)
Supplement: Text S1 — Supporting information text. Included in the supporting text are the details for the parametric analysis of the swimming variables, more detailed methods detailing the model, as well as, the annotated swimming and diving code. (DOCX) [file pcbi.1002792.s004.docx]

**Parametric Analysis of the Swimming**

Considering that there was variation in the experimentally measured parameters, as shown in **Table 1**, the key parameters related to swimming such as the mass of the beetle (M), length of the body (*Lb*), and submerged depth (*h*) were perturbed within the magnitude of their standard deviation, as shown in **Table S1**. The effective area of the middle legs with the laminae extended (Sm+) and the effective area of the hind legs with the laminae extended (Sh+), were perturbed by the standard deviation of the effective area of the legs without the laminae extended, Sm- and Sh- respectively, since these values were used to calculate Sm+ and Sh+. Also, since changes in the submerged depth would result in a change in both the frontal area (*Ay*) and the side area (*Ax*) of the beetle, these two parameters were also perturbed by the same amount as the submerged depth. Further, to determine the effect of the drag on the body (*Cdb*), legs (*Cdl*), and the effect of rotational drag (*Cdr*), these terms were perturbed by ± 10%. After varying these parameters by the specified standard deviations (Perturbed amount in **Table S1**), the results of three leg beating simulations, straight (*hr+hl*, *mr+ml*), S-shaped (*hr*+*hl*,*mr*,*hr*+*hl*,*ml*), and circular (*mr*,*hr*) trajectories were analyzed, and the data recorded in **Table S1**. Considering that there was a wide variation in the perturbed amount between the different parameters, the % change in maximum speed, distance per leg beat, and angular velocity were normalized to determine the relative change per % perturbed (% change / perturbed amount).

Based on the simulation data, the body length and rotational drag only affected the angular velocity of the beetle, with a 5.9% change in body length resulting in a 13.08% change in the angular velocity. This was the largest difference in all of the parameters analyzed, with a normalized value of 2.21. In contrast, although the rotational drag only affected the angular velocity, the normalized value of change was on the same order of magnitude change as changes in the submerged depth, drag coefficient of the body, and drag coefficient of the legs ~0.5. The parameter with the largest experimental variation, and thus largest perturbed amount, body mass, had the least impact in all of the test cases, with an average normalized value for all three cases of 0.19 for the maximum speed, and 0.017 for the distance per beat. When comparing the effect of changes to the effective area of the hind legs and the middle legs, the normalized values indicated that perturbation to the hind legs had an effect 6.95 times larger than changes to the middle legs at the same magnitude, when considering the maximum speed. Similarly, when considering the cases with an equal number of hind and middle leg beats, changes in the hind leg area resulted in 2.46 times larger changes to the distance traveled per leg beat, compared to a change of equal magnitude in the middle legs. The larger impact of the hind legs compared to the middle legs was expected, as described in the earlier analysis on the swimming simulations. The parameter with the largest impact on both the maximum speed and the distance traveled per leg beat was the drag coefficient of the legs (*Cdl*). This makes sense when considering that changes in this term affect both pairs of legs, while the changes in Sm+ and Sh+ only affect one pair of legs at a time. In general, this parameter analysis has demonstrated that a maximal variation in the experimental measurements obtained in this study will lead to a change of ~ 6% in the simulations conducted, demonstrating that within these parameter ranges, the simulation results are valid.

**Supplementary Methods**

***Modeling the swimming dynamics***

The force on a moving object due to the fluid drag is proportional to the dynamic pressure *ρv2* generated by the relative motion of the object with respect to the fluid, i.e.,, where *FD* is the force of drag, *v* is the speed of the object relative to the fluid, *S* is the effective area in contact with the fluid in the direction of movement, and *C­d* is the drag coefficient, a quantity that depends on the shape of the object 1-3. The reference area *S* is defined as the area of the orthographic projection of the object on a plane perpendicular to the direction of motion. For a whirligig beetle swimming on the water surface, the reference area *S* used to calculate the propulsive force is the effective area of the driving legs projected in the *x*, *y*, and *z* directions as shown in **Figure 2B-C**, i.e., *Sx*, *Sy*, and *Sz*.

Assuming that a beetle swims at a speed of *U* and the angular velocity of its body in the *x-y* plane is *ω*, the legs will generate a propulsive force component in the longitudinal direction to propel it forward (**Figure S1A**). The force balance in the *x* and *y* directions while rowing the two middle and hind legs can be described by

(1)

(2)

where *C­dl* is the drag coefficient of legs, and *fr* is the resistance of water. *Sx* and *Sy* are the calculated areas in the *x* and *y* directions based on the angle *Ø* of the leg relative to the center of mass of the body at each time interval, respectively. For instance, the area of the middle leg in the *x* direction (*Smx*) at the maximum angle, *Ømmax*, is equal to *Sm*·sin(*Ømmax* - 90°). In the current model, *Ø* is assumed to change uniformly with time, to avoid complications associated with a non-linear change in angle during the beating motion. To determine the area of the legs in the *y* direction (*Sy*) the same procedure is used; however, the relationship is changed due to the direction of the motion, and *Sy* will be equal to *Sm*·cos(*Ø*-90°). Note that in all variables described from **Eqs**. (1) and (2) on, the subscript ‘*m*’ means the middle leg, ‘*h*’ indicates the hind leg, ‘*r*’ represents ‘right’, and ‘*l*’ denotes ‘left’. The coefficient of drag for the legs (Cdl), which are considered as a plate perpendicular to the flow, is ~1.0 4. Since the directions of the lateral force components by the right and left legs are opposite, they will cancel each other out if beating synchronously. The fluid resistance *fr* corresponds to the force produced by the water opposing the direction of motion in which the beetle is traveling. This resistance to the body is calculated by

$f_{rx}=-\frac{1}{2}sgn(U_{x})\rho U_{x}^{2}C_{db}A_{x};f_{ry}=-\frac{1}{2}\rho U_{y}^{2}C_{db}A_{y}$ (3)

where *Ax*, *Ay* are the lateral (side) and frontal area of the body in contact with the water, and *C­db* is the drag coefficient of the body. In this study *C­db* was approximated as 0.39, a value obtained from previous studies where the drag on whirligig beetles was experimentally measured 5.

While the beetle is moving forward, a torque will be generated with respect to the center of mass to control the turning of the beetle’s body. The effective torque will be

(4)

where *rh* is the distance from the center of mass to the acting point of drag on the hind leg, *Øh* is the angle between the lever arm of the force and the negative *y* axis, and *rrx* is the lever arm of the water resistance. The last term in **Eq**.(4) can be removed, if we assume that the center of mass coincides with the acting point of the lateral resistance in the middle of the body, i.e., *rrx* = 0. Due to the water drag, a resistive torque *Tr* opposite to the direction of rotation will counteract the torque *Tz*.

(5)

where *ω* is the angular rate of the body in the *x*-*y* plane around the *z* axis, and *Lb* is the body length.

When the beetle turns with an angle *β*, we can use the body coordinate system to simplify the dynamics modeling, as shown in **Figure S1(B)**. An assumption that *β* is equal to zero when the beetle is moving in the direction of the *y* axis, and is positive when the beetle turns counterclockwise is made. Over the time interval Δ*t*, the beetle will move from (*x*, *y*, *β*, *t*) to (*x’*, *y’*, *β’*, *t*+Δ*t*). Then, the new coordinates can be calculated as

$x^{'}=x+\Delta x\cdot cos\beta-\Delta y\cdot sin\beta$ (6)

$y^{'}=y+\Delta x\cdot sin\beta+\Delta y\cdot cos\beta$ (7)

$\beta^{'}=\beta+\Delta\beta$ (8)

where Δ*x* and Δ*y* are the increments of displacement in the lateral and longitudinal directions, and Δ*β* is the increment of the rotational angle of the body. Δ*y* should always be positive, while Δ*x* is negative when the beetle moves to the left.

The net torque will cause an angular acceleration in the *x-y* plane, i.e.,, where *Iz* is the mass moment of inertia of the whirligig beetle’s body with respect to the *z* axis, *Tz* is the torque about the *z* axis, and *Tr* is the resistive torque. Next, a dynamic model for the diving process is proposed.

***Modeling the diving dynamics***

The mechanism used by insects with rigid bodies to dive across the air-water interface remains poorly understood in the open literature. Because the distance traveled by the driving legs in the vertical directions is smaller than that in the forward direction during a leg stroke, the leg speeds in the vertical directions are smaller than that in the forward direction. The effective area of driving legs for propulsion in the vertical directions is also smaller than that in the forward direction. Therefore, the force generated by the rowing legs in the forward direction is larger than the component in the vertical direction, and the water resistance is the smallest in the forward direction. So, beetles need to change their direction of motion from the forward direction to the vertical direction in order to dive downwards efficiently and successfully. The curvature force due to the surface tension is proportional to the length of the contact line, so the beetle also needs to minimize the contact line length *C* by rotating its body. In the diving process, priority is given to the rotation of the body in the *y-z* plane, where the forces *fhz*, *fhy* and *frz* will generate a torque in the counterclockwise direction. To turn the body clockwise, the force *fry* needs to be large enough, so that the net torque will be in the clockwise direction, i.e., *Tx* < 0. To this end, either the horizontal forward speed *Uy* or the frontal contact area *Ay* projected in the *y* direction should increase, which requires an instantaneous increase in speed or the formation of the gravity-capillary wave, as shown in **Figure 5**. Since the force *fry* is proportional to *Uy2*, the increase in horizontal forward speed *Uy* is more effective than the increase in frontal contact area *Ay*.

During the pre-diving stage in **Figure S2A**, the tilt angle *γ* and the vertical speed component *Uz* are close to zero. Further, the whirligig beetle beats both the hind legs and middle legs alternately to obtain a longitudinal speed *Uy*. The force components acting on the driving legs in the *y* and negative *z* direction will generate a counterclockwise torque and lead to the oscillation of the body in the *y-z* plane with a tilt angle *γ* (**Figure S2B**). Following each stroke, the beetle’s head will rise up due to the torque generated. Because of the displacement of the anterior part and the posterior part of the body relative to the water, the anterior part of the body will be subject to a downward surface capillary force, and the body’s posterior part will be subject to an upward buoyancy force. Consequently, the body will be subjected to a clockwise torque and the head will go down. When the beetle’s head reaches its lowest point, the head will submerge into the water, followed immediately by the next stroke of the legs (see **Video S2**). Since the contact area *Ay* is maximized at this moment and the length of the lever arm of the force *fry* also attains a maximum, rapid leg beating will generate a net clockwise torque. The net torque will cause the beetle’s body to rotate clockwise towards the upright position. As shown in **Figure S2B**, the net torque is equal to

$\begin{matrix} T_{x}=f_{hz}\cdot r_{h}\sinØ_{h}+f_{hy}\cdot r_{h}\cosØ_{h}+f_{mz}\cdot r_{m}\sinØ_{m}+f_{my}\cdot r_{m}\cosØ_{m}+f_{rz}\cdot r_{rz}-f_{ry}\cdot r_{ry} \\ =\rho C_{dl}\{r_{h}(v_{hz}^{2}S_{hz}\sinØ_{h}+v_{hy}^{2}S_{hy}\cosØ_{h})+r_{m}(v_{mz}^{2}S_{mz}\sinØ_{m}+v_{my}^{2}S_{my}\cosØ_{m})\} \\ +\frac{1}{2}\rho C_{db}(U_{z}^{2}A_{z}r_{rz}-U_{y}^{2}A_{y}r_{ry}) \end{matrix}$ (9)

where *vh*, *vm* are the striking speeds of the hind and middle legs, and *rrz*, *rry* are the lever arms of the corresponding forces. Here, *fry* is just the fluid resistance, and *rry* can be calculated from the height Δ*h* from the center of mass to the ventral side, the rotational angle *γ* of the body, and the position *z* of the beetle’s center of mass. Since the depth *h* of the body under the free surface is equal to *z*+Δ*h*·*cosγ*+*Lb*/2·*sinγ*, then *rry*=*h*/2–*z*. The definitions of the other parameters can be found in **Table 3**.

As shown in **Figure S2B**, the beetle’s body will rotate in the *y-z* plane with respect to the center of mass after each leg stroke. Due to the fluid resistance, a resistive torque *Trd* opposite to the direction of rotation will counteract the torque *Tx*, i.e.,

(10)

where  is the angular rate of the body around the *x* axis, *Lb* is the body length, and *Wb* is the body width. Here, the body of whirligig beetles is approximated as an ellipse.

The instantaneous angular acceleration with respect to the *x* axis in the *y-z* plane can be calculated by, where *Ix* is the mass moment of inertia of the whirligig beetle’s body with respect to the *x* axis. In the body coordinate system, the speed component perpendicular to the forward direction is close to zero, i.e., $U_{y}\sin\gamma\approx U_{z}\cos\gamma$. From *Tx* + *Trd* < *0,* wecan derive a lower bound of *Uy* for successful diving.

The acceleration of the body in the *y* and *z* directions are

(11)

. (12)

As indicated in the *Analysis of Diving Simulation* section of the main text, *Fc* + *Fb*, has been simplified into the segment function, $f_{seg}=\left\{ \begin{aligned} {1.272x10}^{-5}\ln(300(0.0633-t)), 0<t<0.06 \\ {-1.144x10}^{-5}\ln\left( 700\left( t-0.0601 \right) \right), 0.06\leq t\leq0.09 \end{aligned} \right.$ , to account for the slow change of the tilt angle over the first 60 ms of the diving process, followed by the rapid change during the final 29 ms. To determine the constantly changing values of *Ay* and *Az* over time, the tilt angle (γ) was used to determine the tilt of the body at a given time, where *Ay(t)* = *Az(t)* sin γ(t) and *Az(t)* = *Az* cos γ(t). This relationship is described in greater detail in the following section where a simplified version of the Matlab code used in the simulations has been annotated.

**Annotated Code**

*Swimming Code*

The following sets the sampling period, time for the power phase of a leg stroke, time for the return phase of a leg stroke, total run time for the simulation, and the number of leg strokes respectively.

T = 0.01;

t1 = T : T : .05;

t2 = max(t1)+T : T : .1;

t = T : T : 2;

itr_size = (max(t)/max(t2));

A matrix of 1’s and 0’s multiplied by the appropriate constant is used to input the velocity, angular change, and the effective length of each one of the legs individually by the time defined in the parameters above according to the desired leg beating pattern. For example, the hind right leg’s velocity, angular change, and effective length for the circular trajectory matching the beating pattern (mr, hr) are shown below.

tmp = [zeros(size(t1)) ones(size(t2))*.3 ...

zeros(size(t1)) ones(size(t2)) *.3];

vhry = tmp;

for ii = 1 : itr_size/2

vhry = [vhry tmp];

end

tmp = 80*$\pi$/180 : -(80-0)*$\pi$ /180/(length(t1)-1) : 0*$\pi$ /180;

tmp = [zeros(size(t1)) tmp...

zeros(size(t1)) tmp];

$\emptyset$hr = tmp;

for ii = 1 : itr_size/2

$\emptyset$hr = [$\emptyset$hr tmp];

end

tmp = 1.8/1000 : -(1.8-2.34)/1000/(length(t1)-1) : 2.34/1000;

tmp = [zeros(size(t1)) tmp...

zeros(size(t1)) tmp];

rhr = tmp;

for ii = 1 : itr_size/2

rhr = [rhr tmp];

end

The following defines and assigns a value for all of the initial parameters and variables defined in Tables 1-3.

M = 1.0e-5;

Lb = 5.23e-3;

h = 7.4e-4;

$\rho$= 1000;

g = 9.80665;

Ay = 1.31e-6;

Ax = 2.65e-6;

Sm+ = 1.19e-6;

Sh+ = 1.92e-6;

Ux = 0;

Uy = 0;

Cdb = 0.39;

Cdl = 1.0;

Cdr = 1.28;

$\beta$ = (0)*$\pi$/180;

$\omega$ = (0)*$\pi$/180;

$\alpha$ = 0*$\pi$/180;

ax = 0;

ay = 0;

Iz = M * 0.0023^2;

xpos = -0;

ypos = -0;

$\Delta$x = 0;

$\Delta$y = 0;

$\Delta\beta$ = 0;

$\Delta\omega$ = 0;

ii = 1;

The following equations were used to calculate the forces produced by each leg and the resulting fluid resistance in the x and y directions as described by equations 1-3.

%

$f_{rx}=-\frac{1}{2}sgn(U_{x})\rho U_{x}^{2}C_{db}A_{x};f_{ry}=-\frac{1}{2}\rho U_{y}^{2}C_{db}A_{y}$ %

while ii < length(t)

% Force of the middle right leg in x and y %

fmry(ii) = 0.5 * $\rho$ * (vmry(ii) * sin($\emptyset$mr(ii))).^2 * Sm+ * Cdl * sin(($\emptyset$mr (ii));

fmrx(ii) = 0.5 * $\rho$ * (vmry (ii) * cos(($\emptyset$mr (ii))).^2 * Sm+ * Cdl * cos(($\emptyset$mr (ii));

% Force of the middle left leg in x and y %

fmly(ii) = 0.5 * $\rho$ * (vmly (ii) * sin(($\emptyset$ml (ii))).^2 * Sm+ * Cdl * sin($\emptyset$ml (ii));

fmlx(ii) = -0.5 * $\rho$ * (vmly (ii) * cos($\emptyset$ml (ii))).^2 * Sm+ * Cdl * cos($\emptyset$ml (ii));

% Force of the hind right leg in x and y %

fhry(ii) = 0.5 * $\rho$ * (vhry (ii) * sin($\emptyset$hr (ii))).^2 * Sh+ * Cdl * sin($\emptyset$hr (ii));

fhrx(ii) = 0.5 * $\rho$ * (vhry (ii) * cos($\emptyset$hr (ii))).^2 * Sh+ * Cdl * cos($\emptyset$hr (ii));

% Force of the hind left leg in x and y %

fhly(ii) = 0.5 * $\rho$ * (vhly (ii) * sin($\emptyset$hl (ii))).^2 * Sh+ * Cdl * sin($\emptyset$hl (ii));

fhlx(ii) = -0.5 * $\rho$ * (vhly (ii) * cos($\emptyset$hl (ii))).^2 * Sh+ * Cdl * cos($\emptyset$hl (ii));

% Fluid resistive force in x and y %

fry(ii) = - Cdb * Ay * $\rho$ * (Uy (ii)^2) / 2;

frx(ii) = -sign(Ux (ii)) * Cdb * Ax * $\rho$ * (Ux (ii)^2) / 2;

The following set of equations is used to calculate the acceleration of the body in the x and y directions as described by equations 1 and 2 above.

% Acceleration of the body in x and y %

ay (ii) = (fmry (ii) + fmly (ii) + fhry (ii) + fhly (ii) + fry (ii)) / M;

ax (ii) = (fmrx (ii) + fmlx (ii) + fhrx (ii) + fhlx (ii) + frx (ii)) / M;

The torque generated by all of the legs and the resistive torque are calculated according to equations 4 and 5 as shown below.

%

%

Tz(ii) = fmry (ii) * rmr(ii) * sin($\emptyset$mr (ii)) + fmrx (ii) * rmr (ii) * cos($\emptyset$mr (ii)) - ...

fmly (ii) * rml (ii) * sin($\emptyset$ml (ii)) + fmlx (ii) * rml (ii) * cos($\emptyset$ml (ii)) + ...

fhry (ii) * rhr (ii) * sin($\emptyset$hr (ii)) + fhrx (ii) * rhr (ii) * cos($\emptyset$hr (ii)) - ...

fhly (ii) * rhl (ii) * sin($\emptyset$hl (ii)) + fhlx (ii) * rhl (ii) * cos($\emptyset$hl (ii));

Tr(ii) = -sign($\omega$ (ii)) * $\rho$ * Cdr * h * $\omega$ (ii)^2 * Lb ^4 / 64;

Angular acceleration, body velocity in the x and y directions, change in body angular velocity, change in body turning angle, and change in body position are all calculated based on the following equations 6-8 along with the ones previously described above.

% Angular acceleration %

$\alpha$ (ii) = (Th (ii) + Tr (ii))*1 / Iz;

% Velocity of the body in x and y %

Ux (ii+1) = Ux (ii) + ax (ii) * T;

Uy (ii+1) = Uy (ii) + ay (ii) * T;

% Change in angular velocity over time %

$\Delta\omega$ (ii) = $\alpha$ (ii) * T;

$\omega$ (ii+1) = $\omega$ (ii) + $\Delta\omega$ (ii);

% Change in the rotational angle over time %

$\Delta\beta$ (ii) = ($\omega$ (ii) + $\omega$ (ii+1)) * T / 2;

$\beta$ (ii+1) = $\beta$ (ii) + $\Delta\beta$ (ii);

% Change in x and y body position %

$\Delta$x (ii) = (Ux (ii) + Ux (ii+1)) / 2 * T;

$\Delta$y (ii) = (Uy (ii) + Uy (ii+1)) / 2 * T;

ypos (ii+1) = ypos (ii) + $\Delta$y (ii) * cos($\beta$ (ii)) + $\Delta$x (ii) * sin($\beta$ (ii));

xpos (ii+1) = xpos (ii) - $\Delta$y (ii) * sin($\beta$ (ii)) + $\Delta$x (ii) * cos($\beta$ (ii));

ii = ii + 1;

end

*Diving Code*

The following sets the sampling period, time for the power phase of a leg stroke, time for the return phase of a leg stroke, total time the simulation runs, and the number of leg strokes respectively.

T = 0.0015;

t1 = T : T : .0075;

t2 = max(t1)+T : T : .015;

t = T : T : .09;

itr_size = (max(t)/max(t2));

A matrix of 1’s and 0’s multiplied by the appropriate constant is used to input the velocity, angular change, and the effective length of each one of the legs individually by the time defined in the parameters above according to the desired leg beating pattern. For example, the hind leg’s velocity, angular change, and effective length for the trajectory matching the average beating parameters are shown below.

vhy = [1 1 1 1 1 0 0 0 0 0 0 1 1 1 1 1 0 0 0 0 0 0 1 1 1 1 1 0 0 0 0 0 1 1 1 1 1 0 0 0 0 0 0 1 1 1 1 1 0 0 0 0 1 1 1 1 1 0 0 0]*0.18;

tmp = 83.53*$\pi$/180 : -(83.53-33.04)*$\pi$ /180/(length(t1)-1) : 33.04*$\pi$ /180;

tmp = [tmp zeros(size(t2))];

$\emptyset$h = tmp;

for ii = 1 : itr_size

$\emptyset$h = [$\emptyset$h tmp];

end

tmp = 1.7/1000 : -(1.7-2.3)/1000/(length(t1)-1) : 2.3/1000;

tmp = [tmp zeros(size(t2))];

rh = tmp;

for ii = 1 : itr_size

rh = [rh tmp];

end

The following defines and assigns a value for all of the initial parameters and variables defined in Tables 1-3.

M = 1.0e-5;

Lb = 5.23e-3;

Wb = 2.2e-3;

$\rho$ = 1000;

g = 9.80665;

t3 = T : T : .06;

t4 = max(t3)+T : T : .09;

$\Delta$mg1 = (1+(0.43-3*t3).^2)*M*g - M*g; (% Initial conditions of segment function %)

$\Delta$mg2 = (1-(20*t4-1.08).^2)*M*g - M*g; (% Latter conditions of segment function %)

$f_{seg}$ = [$\Delta$mg1 $\Delta$mg2];

Cdb = 0.39;

Cdl = 1.0;

Cdr = 1.28;

Ay = 1.31e-6;

Az = Sb = 7.32e-6;

Sm+ = 1.19e-6;

Sh+ = 1.92e-6;

Uz = 0;

Uy = Upre = 0.1;

$\omega$ = (-333)*$\pi$ /180;

$\gamma$ = (-7)*$\pi$ /180;

$\alpha$ = 0*$\pi$ /180;

az = 0;

ay = 0;

zpos = 0;

ypos = 0;

$\Delta$z = 0;

$\Delta$y = 0;

$\Delta\gamma$ = 0;

$\Delta\omega$ = 0;

$\Delta$h = 0.74e-3;

$\Delta$h’ = 0;

Ix = 1.5250e-10;

ii = 1;

The following equations were used to calculate the area in y based on its relationship with the angle change and the area in z along with the forces produced by each leg and the resulting fluid resistance and acceleration of the body in the y and z directions as described by equations 11 and 12.

%

%

while ii < length(t)

% Change in the frontal area during diving %

Ay (ii) = abs(Az * sin($\gamma$ (ii)));

% Force of the middle legs in y and z %

fmy(ii) = 0.5 * $\rho$ * (vmy (ii)*sin($\emptyset$m (ii)))^2 * Sm+ * Cdl * sin($\emptyset$m (ii));

fmz(ii) = -0.5 * $\rho$ * (vmy (ii)*cos($\emptyset$m (ii)))^2 * Sm+ * Cdl * cos($\emptyset$m (ii));

% Force of the hind legs in y and z %

fhy(ii) = 0.5 * $\rho$ * (vhy (ii)*sin($\emptyset$h (ii))).^2 * Sh+ * Cdl * sin($\emptyset$h (ii));

fhz(ii) = -0.5 * $\rho$ * (vhy (ii)*cos($\emptyset$h (ii))).^2 * Sh+ * Cdl * cos($\emptyset$h (ii));

% Resistive force of the body in y and z %

fry(ii) = - Cdb * Ay (ii) * $\rho$ * ((Uy (ii)*cos($\gamma$ (ii)) - Uz (ii)*sin($\gamma$ (ii)))^2) / 2;

frz(ii) = Cdb * Az * cos($\gamma$ (ii)) * $\rho$ * ((Uy (ii)*sin($\gamma$ (ii)) + Uz (ii)*cos($\gamma$ (ii)))^2) / 2;

% Sum of the y and z forces %

fy(ii) = fmy (ii) * 2 + fhy (ii) * 2 + fry (ii) * cos($\gamma$ (ii)) + frz (ii) * sin($\gamma$ (ii)) + $f_{seg}$ (ii) * sin($\gamma$ (ii));

fz(ii) = fhz (ii) * 2 + fmz (ii) * 2 - fry (ii) * sin($\gamma$ (ii)) + frz (ii) * cos($\gamma$ (ii)) + $f_{seg}$ (ii) * cos($\gamma$ (ii));

% Acceleration of the body in y and z %

ay (ii) = fy (ii) / M;

az (ii) = fz (ii) / M;

The torque generated by all of the legs and the resistive torque opposite to the direction of rotation along with the effective length of the lever arm in which this force is applied are calculated according to equations 9 and 10 as shown below.

% $\begin{matrix} T_{x}=f_{hz}\cdot r_{h}\sinØ_{h}+f_{hy}\cdot r_{h}\cosØ_{h}+f_{mz}\cdot r_{m}\sinØ_{m}+f_{my}\cdot r_{m}\cosØ_{m}+f_{rz}\cdot r_{rz}-f_{ry}\cdot r_{ry} \\ =\rho C_{dl}\{r_{h}(v_{hz}^{2}S_{hz}\sinØ_{h}+v_{hy}^{2}S_{hy}\cosØ_{h})+r_{m}(v_{mz}^{2}S_{mz}\sinØ_{m}+v_{my}^{2}S_{my}\cosØ_{m})\} \\ +\frac{1}{2}\rho C_{db}(U_{z}^{2}A_{z}r_{rz}-U_{y}^{2}A_{y}r_{ry}) \end{matrix}$

%

% Torque generated by all of the legs about the x axis %

Txl(ii) = fhy (ii) * rh (ii) * sin($\emptyset$h (ii)) - fhz (ii) * rh (ii) * cos($\emptyset$h (ii)) + fmy (ii) * rm (ii) * sin($\emptyset$m (ii)) - fmz (ii) * rm (ii) * cos($\emptyset$m (ii));

% Effective length of the lever arm %

rxr(ii) = (abs(0.5 * Lb * sin($\gamma$ (ii))) + abs($\Delta$h * cos($\gamma$ (ii))) - abs($\Delta$h’ (ii))) / 2;

if abs(rxr (ii)) > abs($\Delta$h * cos($\gamma$ (ii)))

rxr (ii) = abs($\Delta$h * cos($\gamma$ (ii)));

end

% Resistive torque %

Txr(ii) = -abs(fry(ii) * abs(rxr (ii)));

% Sum of Torque %

Tx(ii) = Txl(ii) + Txr(ii);

Trd(ii) = 0;

for jj = 1 : ((Lb /2) / (Lb /200))

% Resistive torque opposing the direction of rotation %

Trd(ii) = Trd (ii) + abs(2 * sign($\omega$ (ii)) * 0.5 * $\rho$ * (jj* Lb /200) * ($\omega$ (ii)*jj* Lb /200)^2 * Cdr * 0.5 * Wb * sqrt(1-(jj/100)^2) * Lb /200);

end

Trd (ii) = - Trd (ii)*sign($\omega$ (ii));

The angular acceleration, body velocity in the y and z directions, change in body angular velocity, change in tilt angle, and change in body position are all calculated by the following equation and those previously listed above, 11 and 12.

% Angular acceleration %

α(ii) = (Tx(ii) + Trd (ii)) / Ix;

% Change in velocity in y and z over time %

Uz (ii+1) = Uz (ii) + az (ii) * T;

Uy (ii+1) = Uy (ii) + ay (ii) * T;

% Change in angular velocity over time %

$\Delta\omega$ (ii) = $\alpha$ (ii) * T;

$\omega$ (ii+1) = $\omega$ (ii) + $\Delta\omega$ (ii);

% Change in tilt angle over time %

$\Delta\gamma$ (ii) = ($\omega$ (ii) + $\omega$ (ii+1)) * T / 2;

$\gamma$ (ii+1) = $\gamma$ (ii) + $\Delta\gamma$ (ii);

% Change in body position %

$\Delta$z (ii) = (Uz (ii) + Uz (ii+1)) / 2 * T;

$\Delta$y (ii) = (Uy (ii) + Uy (ii+1)) / 2 * T;

$\Delta$h’ (ii+1) = $\Delta$h’ (ii) + abs($\Delta$y (ii) * sin($\gamma$ (ii))) + abs($\Delta$z (ii) * cos($\gamma$ (ii)));

ypos (ii+1) = ypos (ii) + $\Delta$y (ii) * cos($\gamma$ (ii))/1 - $\Delta$z (ii) * sin($\gamma$ (ii));

zpos (ii+1) = zpos (ii) + $\Delta$y (ii) * sin($\gamma$ (ii))/1 + $\Delta$z (ii) * cos($\gamma$ (ii));

ii = ii + 1;

end

**Supplementary References:**

1 Whitehead, A. N. R., Bertrand. Principia Mathematica. *Cambridge: Cambridge University Press.* **2** (1925).

2 Denny, M. W. Air and Water: The Biology and Physics of Life’s Media. *Princeton: Princeton University Press* (1993).

3 Voise, J. & Casas, J. The management of fluid and wave resistances by whirligig beetles. *Journal of The Royal Society Interface* **7**, 343-352, doi:10.1098/rsif.2009.0210 (2010).

4 Frederic P. Miller, A. F. V., John McBrewster. Drag Coefficient. *Alphascript Publishing* (2010).

5 Nachtigall, W. Hydromechanics and biology. *European Biophysics Journal* **8**, 1-22, doi:10.1007/bf01047102 (1981).
